# First molecular examination of Vietnamese mudflat snails in the genus *Naranjia* Golding, Ponder & Byrne, 2007 (Gastropoda: Amphibolidae)

**DOI:** 10.1038/s41598-020-75200-8

**Published:** 2020-10-30

**Authors:** Takumi Saito, Larisa Prozorova, Ngo Xuan Quang, Satoshi Chiba

**Affiliations:** 1 Graduate School of Life Science, Tohoku University, Sendai, Miyagi 980-0845 Japan; 2 Department of Biology, Faculty of Science, Toho University, Funabashi, Chiba Japan; 3 Federal Scientific Center of the East Asia Terrestrial Biodiversity, Far Eastern Branch of Russian Academy of Sciences, Vladivostok, Russia; 4 Graduate University of Science and Technology, Vietnam Academy of Science and Technology, 18, Hoang Quoc Viet, Cau Giay, Ha Noi, Vietnam; 5 Department of Environmental Management and Technology, Institute of Tropical Biology, Vietnam Academy of Science and Technology, 85 Tran Quoc Toan Street, District 3, Ho Chi Minh City, Vietnam; 6 Center for Northeast Asian Studies, Tohoku University, Sendai, Miyagi Japan

**Keywords:** Biodiversity, Conservation biology, Taxonomy

## Abstract

Maritime ecosystems in Vietnam such as mangroves and mud flats are characterized by high biodiversity. However, elements of its biodiversity remain unclear and highly threatened. In this context, the assessment of rare species is a starting point to develop effective strategies for the conservation of entire ecosystems. In this paper, we report upon cryptic amphibolid gastropods in Vietnamese mangrove forests from the Mekong Delta. The snail fauna in the mangrove forests was previously known from published literature and three museum specimens as three amphibolid species, ‘*Amphibola*’ *burmana*, *A. quadrasi*, *Salinator fragilis* and ‘*S.*’ *quadrasi*. We investigated the identities of such snails using molecular and morphological methods. The amphibolids found in this survey were identified to belong to the genus *Naranjia,* new for Vietnam fauna. In addition, our phylogenetic analyses suggested that the Vietnamese amphibolids were the same species as *Naranjia* sp. reported from Thailand, and the amphibolids have both genetic and morphological polymorphisms within the population. These findings add to the great biodiversity of Vietnamese mangrove forests and mudflats.

## Introduction

The country of Vietnam straddles some 13 degrees of latitude with a corresponding long coastline with biodiversity hotspot which are highly threatened^1–6^. In particular, mangrove forests which mainly occur in the Mekong Delta along the southern coastline, and also around the Red River Delta in the north, are being destroyed rapidly in spite of their importance to economics, coastal stability, and habitat for organisms^7–9^. Within the Mekong Delta, mangrove forests are particularly developed in Ca Mau and Bac Lieu provinces^9^. Besides these areas, a mangrove belt still covers part of the shoreline in Kien Giang Province also in the Mekong Delta, belonging to Kien Giang Biosphere Reserve, recognized by UNESCO in 2006^10–12^. Though molluscs are known as one of the most diverse, abundant and important components of the mangrove ecosystem, the estuarine malacofauna of Vietnam, especially in the Mekong Delta is still poorly studied^13–15^. In the delta, several molluscan species occurring in the Ca Mau mangroves were reported by N. Thach in his general monographs on Vietnam molluscan fauna^16–18^, and three other additional species were recorded from the Ba Tai tidal mangrove forest in Kien Luong District of the Kien Giang Province^19, 20^.

Our first malacological survey in 2017 encompassing the mangrove forests, tidal mudflats, and estuaries along the coastline of Kien Giang Province revealed the presence of more than 60 gastropod and bivalve species in the Ba Tai tidal mangrove forest (An Binh Commune, Kien Luong District), which is recognized as a molluscan diversity hot spot^21^. The forest growing between the limestone Ba Tai hills and brackish water Ba Tai Lake belongs to the Kien Luong Protected Area which encompasses 200 km of low coastline and supports one of the last significant areas of the natural mangroves and mud flats in Kien Giang Province^12^. In order to further understand the molluscan components of the Mekong Delta, the second field survey was undertaken in the same mangrove forest by an international research team in May 2018. As a result, some rare and new records for estuarine Vietnamese molluscan taxa including those Amphibolidae were recorded^22^. These findings are of great importance in the absence of comprehensive biodiversity data on coastal ecosystems in Vietnam. The assessment of rare species is useful in the development of effective conservation strategies for entire ecosystems^23–25^.

**Figure 1:**
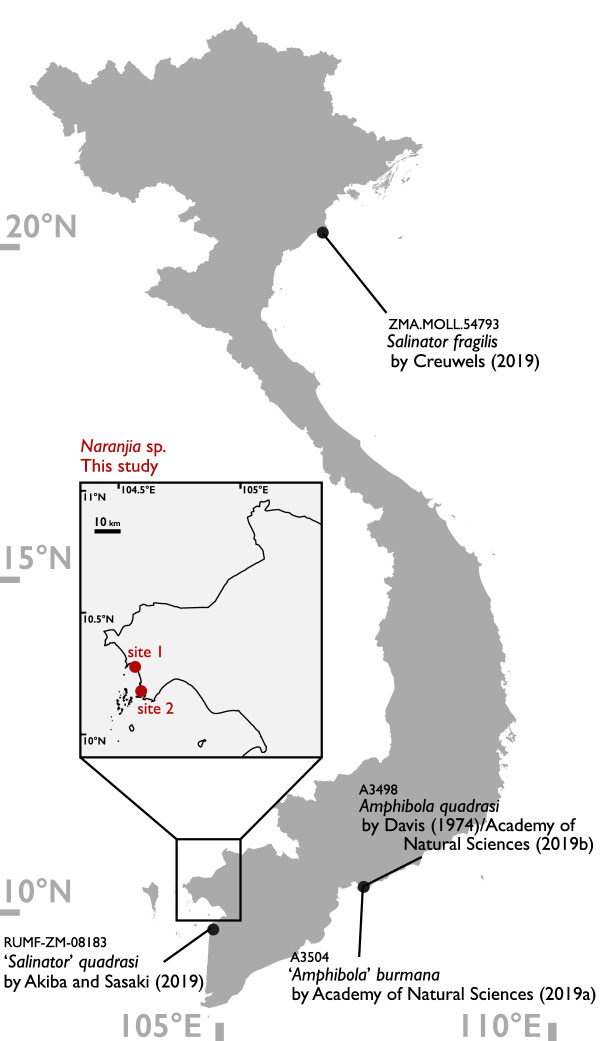
Locality map of Vietnamese Amphibolidae. Red circles indicate localities (see Fig. 2) where amphibolids were collected in this study. Black circles indicate localities where amphibolids were recorded based on published literature and museum collections. The reference numbers above the scientific names indicate the catalogue number of each specimen. This map was created from GADM database, version 3.4^45^using QGIS version 2.18^46^.

Amphibolid snails are an air-breathing taxon that inhabit mangrove forests, mudflats and salt marshes^26^. This family is poorly studied in Vietnam. In fact, no recent Vietnamese molluscan monographs have recorded this family^16–18, 27^. Thus, Vietnamese amphibolids have not been examined in morphological studies involving the taxonomic revision of the superfamily Amphiboloidea^28, 29^. However, there is a single record^30^ in the literature and the three GBIF online records as well as one lot in the malacology collection in the Academy of Natural Science, Philadelphia (ANSP)^31–34^ (Fig. 1; three species, ‘*Amphibola*’ *burmana*, *A. quadrasi*, *Salinator fragilis* and ‘*S.*’ *quadrasi*). Note that the ANSP record may refer to the same material as those reported upon by Davis (1974) because the locality name, collector, collection date and station name were all the same. In this study, we report details of the occurrence of these rare mangrove amphibolid species and examine for the first time the molecular sequences of these rare mangal amphibolid snails in Vietnam.

## Material and methods

**Figure 2:**
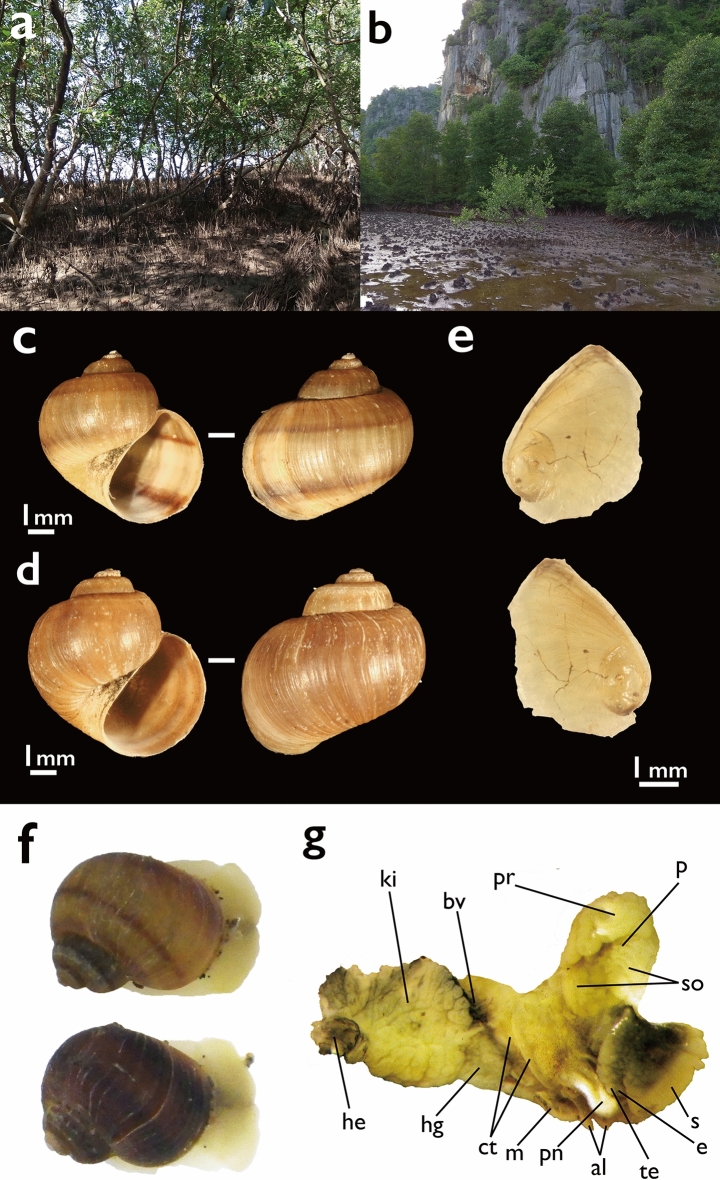
(**a**) The environment of the locality; site 1, mangrove forest near Ha Tien Town. (**b**) The environment of the locality; site 2, Ba Tai mangroves. (**c**) Shell morphology of *Naranjia* sp. (Specimen No. ScNM487). This specimen was collected from site 1 (Fig. 1). Scale bar = 1 mm. Shell length (SL) is around 6.6 mm. Deposited in Tohoku University. (**d**) Shell morphology of *Naranjia* sp. (Specimen No. ScNM488). This specimen was collected from site 1 (Fig. 1). Scale bar = 1 mm. SL is around 7.2 mm. Deposited in Tohoku University. (**e**) Operculum of *Naranjia* sp. (Specimen No. ScNM488). Above: the front side, below: the back side. Scale bar = 1 mm. Deposited in Tohoku University. (**f**) Live specimens of *Naranjia* sp. from site 1. Molecular data of these specimens were not examined. (**g**) Head and organs of mantle cavity of *Naranjia* sp. from site 1. (Specimen No. 96v2018-10/4). Deposited in Federal Scientific Center of the East Asia Terrestrial Biodiversity (FSCEATB). Scale bar = 1 mm. *al* anal lobe, *bv* blood vessel, *ct* opposed ciliary tracts, *e* eye, *he* heart, *hg* hypobranchial gland, *ki* kidney, *m* mantle, *p* papillae on the distal surface of the spermovipositor, *pn* pneumostome, *pr* prostate gland, *s* snout, *so* spermovipositor, *te* tentacle.

Vietnamese amphibolids were first discovered at two sites in the Kien Luong District, Kien Giang Province on May 3rd 2018^22^. One site was at a mangrove forest near Ha Tien Town (17 specimens collected; Fig. 1: site 1, Fig. 2a), and the other was in the Ba Tai mangroves (one specimen collected; Fig. 1: site 2, Fig. 2b). Molluscs were hand-picked from mud flats and open mangrove forests with *Rhizophora* and *Avicennia* trees. Three individuals from site 1 (ScNM 486, 487 and 488; Fig. 2c–e) were preserved in 99.5% ethanol for molecular analysis and other 14 individuals from site 1 (Federal Scientific Center of the East Asia Terrestrial Biodiversity [FSCEATB] 96v2018-10/1-14; Fig. 2g) were stored in 95% ethanol for morphological study after collecting (for details, see Supplementary Information 1). Total DNA was isolated from each of three individuals by DNeasy Blood & Tissue kit (Qiagen Inc., Hilden, Germany). Then, two mitochondrial (cytochrome oxidase subunit 1 [CO1] and 16S ribosomal DNA [16S]) and one nuclear marker (28S ribosomal DNA [28S]) were amplified according to the methods of Golding^26^. However, an annealing temperature of strictly 63°C was used for 28S instead of 52°C to 63°C^26^. Each amplified gene fragment was sequenced after purification via ExoSAP-IT (Affymetrix Inc., California, USA). Sequencing was performed by Eurofins Genomics (Tokyo, Japan). All sequences were deposited in the GenBank database (LC575065-LC575072). For phylogenetic analysis, each sequence was firstly aligned using the default settings of MUSCLE v3.8.31^35^ with sequences of 26 amphibolid materials and one outgroup material of Golding (2012; dataset B)^26^. Then, to eliminate the uncertainty of the 16S and 28S alignments, trimAl 1.2^36^ was used to select regions of the aligned sequences for analyses (output files: Supplementary Information 2–3). Secondly, to select an appropriate evolutionary model and partitioning schemes of each region, we used PartitionFinder 2^37^ with Akaike's information criterion with correction for small sample size (AICc). Based on selected evolutionary models and partitioning schemes by PartitionFinder 2 (Supplementary Information 4), phylogenetic trees were obtained by Bayesian inference (BI) and maximum likelihood (ML) methods. BI analysis was conducted using MrBayes5D Version 3.1.2.2012.12.13^38^, which is an extended version of MrBayes 3.1.2^39^, and ML analysis was performed using IQ-TREE version 1.6.7^40^. In MrBayes5D, we used four simultaneous chains for one million generations, and trees were sampled every 100 generations. We discarded the first 1001 trees as burn-in after examining convergence and effective sample size (ESS; Over 200) using Tracer v. 1.6^41^; the remaining samples were used to estimate phylogeny. IQ-TREE was run under the evolutionary models and partitioning schemes selected by PartitionFinder 2 with –spp option (the same set of branch lengths across partitions with allowing each partition to evolve under its rate). Bootstrap analysis was executed using IQ-TREE’s ultra fast bootstrap option (-bb)^42^ with 5000 replicates. In addition to the analysis using the combined three regions, we analysed mitochondrial and nuclear regions respectively using the same methods (for detail parameters: Supplementary Information 5–6). Furthermore, we calculated K2P-corrected pairwise distances between CO1 sequences of *Naranjia* species to compare with Golding^26^ using MEGA 6.06^43^. Finally, to examine the past records in GBIF, we investigated museum specimens with the help of the curators.

## Results and discussion

**Figure 3:**
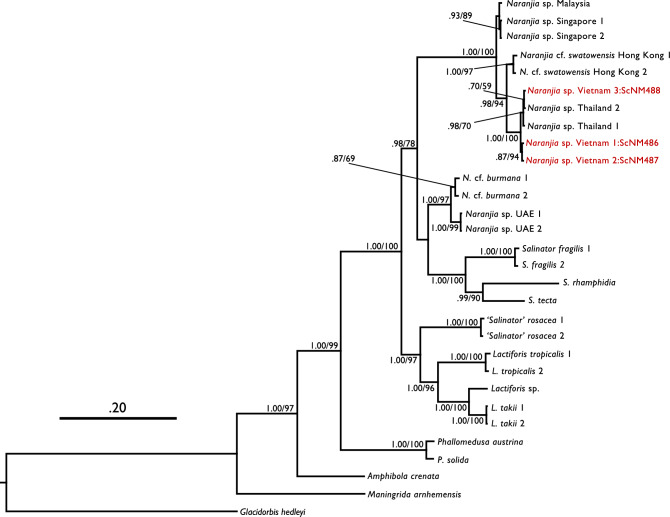
Bayesian phylogenetic tree inferred from a combined dataset of two mtDNA and one ncDNA sequences (mtCO1, mt16S; nc28S; total 2209 bp). Numbers at the branch nodes represent Bayesian posterior probability and Ultrafast bootstrap value in IQtree. Red OTUs indicate Vietnamese amphibolids. Refer to Golding (2012)^26^ for detailed information of other amphibolids.

First, inferred phylogeny showed that Vietnamese amphibolids belonged to the genus *Naranjia* Golding, Ponder & Byrne, 2007 because of fully supported monophyly with two *Naranjia* sp. materials^26^ from Thailand (Fig. 3; Bayesian posterior probability = 1.00/Bootstrap value = 100). Furthermore, this monophyletic group was sister to a clade of *N.* cf. *swatowensis* (Yen, 1939) from Hong Kong, type species of the *Naranjia*^28^ and this monophyletic clade was relatively well-supported (0.98/94). This clade was in turn sister to another clade of *Naranjia* sp. from Malaysia and Singapore and was fully supported (1.00/100). Thus, Vietnamese amphibolids are here regarded as belonging to the genus *Naranjia* on the basis of molecular phylogeny, and this is the first record of the genus *Naranjia* in Vietnam.

In addition to molecular phylogeny, we examined the morphology of the shell, operculum and soft tissue of Vietnamese amphibolids (Fig. 2c–g). The morphology of organs in the mantle cavity is described on the basis of dissection of specimens deposited in FSCEATB. We used terminology following Golding et al.^28, 29^. The morphological treats are as follows: shell globose, diameter to 8 mm, umbilicate, with short spire and large last whorl, nearly smooth; exterior pale-orange to brownish-orange, uniformly coloured or sometimes with one to two dark brown spiral bands; aperture semi-lunar; umbilicus of intermediate width (Fig. 2c,d). Operculum thin, corneous, semi-transparent pale yellow with eccentric, paucispiral nucleus, inner surface of nucleus slightly raised, smooth and bright (Fig. 2e). Foot short, anterior and posterior region slightly convex; head with short, bilobed snout, unpigmented distally and light yellow proximally, separated from foot by deep furrow; cephalic tentacles short, flattened-semicircular; eyes at outer base (Fig. 2f,g). Pneumostome with large white coloured pneumostomal lobe attached to anal lobe on right side of mantle edge; mantle roof with few blood vessels; opposed ciliary tracts not short; kidney on roof of mantle cavity, ovate-triangular, large; hypobranchial gland large triangular, white in ethanol-preserved specimens, located at right anterior corner of mantle cavity roof between kidney and opposed ciliary tracts; prostate gland large, oval, white, formed by a tightly packed tube, uniform for their entire length; large spermovipositor covered by soft finger-shaped papillae on its distal surface (Fig. 2g). These morphological traits were consistent with those of *Naranjia* cf. *swatowensis*^28^, the only *Naranjia* species documented in detail, although we could not examine the central nervous system and radula. In particular, our specimens had shells which were brownish-orange and the spermovipositor distally covered by finger-like papillae, which Golding (2008) indicated as the distinguishing traits of the genus *Naranjia*.

Although statistical support was not high, Vietnamese *Naranjia* materials were divided into two lineages; one was closely related to Thai *Naranjia* sp., and another represented a potentially endemic lineage in Vietnam (Fig. 3). Although 28S sequences from two Thai *Naranjia* species were not included, our mitochondrial phylogeny also indicated similar relationships between the two Vietnamese lineages (Supplementary Information 5). However, K2P pairwise CO1 distances among the Vietnamese individuals were calculated to be 0.61% to 1.70%, and the CO1 distances between Vietnamese and Thai *Naranjia* sp. were 0.46 to 1.54% (Table 1). These values are close to the average intraspecies CO1 distances of amphibolids shown by Golding^26^. Accordingly, the Vietnamese *Naranjia* sp. and Thai *Naranjia* sp. seem to be the same species, and the presence of two lineages can imply a genetic polymorphism. In contrast, all of the CO1 distances between Vietnamese amphibolids and other *Naranjia* species were over 6.60% (Table 1). As Golding (2012) showed that CO1 distances of interspecies were 4.25 to 24.55%, the Vietnamese (and Thai) amphibolids can be considered to be genetically distinct from other *Naranjia* species including *N.* cf. *swatowensis* from Hong Kong. This suggests the existence of cryptic diversity in the Asian *Naranjia* species complex^26^. On the other hand, the shells of Vietnamese specimens display several different colors and band patterns (Fig. 2c,d,f; pale-orange to brownish-orange, no band: five, one band: seven, two bands: five in 17 individuals from site 1). In some Amphibolidae species, phenotypic polymorphism within species was reported^28, 44^. Thus, the Vietnamese amphibolids seem to show not only genetic but also morphological polymorphism such as shell color and band pattern.

**Table 1:** K2P pairwise CO1 distances (%) between Vietnamese *Naranjia* sp. and other *Naranjia* spp.

	ScNM486	ScNM487	ScNM488
*Naranjia* sp. Vietnam 1: ScNM486	–		
*Naranjia* sp. Vietnam 2: ScNM487	0.61%	–	
*Naranjia* sp. Vietnam 3: ScNM488	0.17%	0.17%	–
*N*. cf. *burmana* 1	15.12%	15.51%	15.51%
*N*. cf. *burmana* 2	14.92%	14.92%	15.31%
*N*. cf. *swatowensis* 1	7.65%	7.65%	8.89%
*N*. cf. *swatowensis* 2	6.60%	6.60%	7.46%
*Naranjia* sp. UAE 1	13.80%	13.80%	14.57%
*Naranjia* sp. UAE 2	13.80%	13.80%	14.57%
*Naranjia* sp. Malaysia 1	7.85%	7.85%	8.03%
*Naranjia* sp. Singapore 1	7.14%	7.14%	7.32%
*Naranjia* sp. Singapore 2	7.14%	7.14%	7.32%
*Naranjia* sp. Thailand 1	0.15%	0.15%	0.02%
*Naranjia* sp. Thailand 2	0.15%	0.15%	0.05%

In Vietnam, as mentioned above, there are only four records of amphibolids (Fig. 1), and all of these species (i.e., *burmana*, *fragilis*, *quadrasi*, as either *Amphibola* or *Salinator*) were likely to have been misidentified, as all recorded species are actually not distributed in Vietnam after recent taxonomic revision^28^. We examined museum materials of two amphibolids included in three GBIF records, and one shell of *Amphibola quadrasi* from ANSP collection (Supplementary Information 7). The shell of amphibolids does not have enough information to be identified to species and even genus^28^, and so we could not identify these specimens. Nevertheless, these specimens are included in Salinatorinae (*Lactiforis*, *Naranjia* or *Salinator*) based on shell size and morphology^28^. In addition, some specimens were polymorphic in the shell shape and size in spite of specimens from the same localities (Supplementary Information 7c–e), and it may contain two or more species, which merit further investigation.

Although further researches are needed to reveal the actual distribution of Vietnamese amphibolids, in any case, this paper is the first report of Vietnamese Amphibolidae. Vietnamese amphibolids appear to be rare, because the taxonomic revision of Amphiboloidea^26, 28^, catalogues of Vietnamese mollusks by Thach^16–18, 27^, and checklist of Vietnamese Khanh Hoa mangal molluscan fauna^15^ have not reported upon Amphiboloidea species. In contrast, our findings show that amphibolids are rare but distributed along the Vietnamese coastline sporadically and have genetic and phenotypic polymorphism from one locality. Therefore, immediate assessment of distribution and diversity of Amphiboloidea is required considering the rapid decline in the area of Vietnamese mangrove forests^7, 8^. Amphibolids may be an excellent indicator of the conservation status of cryptic biodiversity in Vietnamese mangrove forests.

### Ethics declarations

All necessary permits for sampling and observational field studies have been obtained by the authors from the Institute of Tropical Biology (Vietnam Academy of Science and Technology) and local competent authorities.

## Supplementary information

Supplementary Information 1.

Supplementary Information 2–7.

## Data Availability

Sequences of *Naranjia* sp. were deposited in the GenBank (CO1:LC575065-LC575067/16S:LC575068-LC575070/28S: LC575071-LC575072). Additional data are available from supplementary materials.
