# Supplementary material for: First molecular examination of Vietnamese mudflat snails in the genus Naranjia Golding, Ponder & Byrne, 2007 (Gastropoda: Amphibolidae)
Source: Sci Rep. 2020 Oct 30;10:18714. doi: 10.1038/s41598-020-75200-8 (PMC7603323; doi:10.1038/s41598-020-75200-8)

Supplementary Information

First molecular examination of Vietnamese mudflat snails: New record of the genus Naranjia Golding, Ponder & Byrne, 2007 (Gastropoda: Amphiboloidea: Amphibolidae) in Vietnam

Takumi Saito*, Larisa Prozorova, Ngo Xuan Quang, Satoshi Chiba

*Corresponding author: saito.zef@gmail.com

Contents

Supplementary Information_1.xslx (other files)

Supplementary Information 1.

Detailed information of material examined new molecular data in this study.

Supplementary Information.pdf

Supplementary Information 2.

16S sequences after eliminating uncertainty of the alignment.

Supplementary Information 3.

28S sequences after eliminating uncertainty of the alignment.

Supplementary Information 4.

Information of partitioning scheme and evolutionary models for phylogenetic analyses.

Supplementary Information 5.

The Bayesian phylogenetic tree inferred from a combined dataset.

Supplementary Information 6.

The Bayesian phylogenetic tree inferred from 28S sequences.

Supplementary Information 7.

The shells of Amphibolidae in the museums.

Supplementary Information 2

>Naranjia_sp_Vietnam_486
AAAACATAGCCCTAAGAAGATATTTAGGGTAAGCCCTGCCCGGTGACGGAAGTTTAACGGCCGCAGTA
CCTTGACTGTGCTAAGGTAGCGTAATCAGTTGGCTTTTAAATGGAGTCTGGTATGAACGGGTCTATGG
GGGAAAGCTGTCTCAGGCTTAGATTTTGAAATTATTAATAAGGTGAAAATACCTTTTGTCAAAAAAAA
GACGAGAAGACCCTTAGAGCTTTTAATAAATTGTAGTATATACAATACTTTTTTGTTGGGGCGACACG
GAGACATTGAACTCTCTCTAATTTAAATTACTTGCCGACTTTTTGTAAGTAAGAATAAGCTACCTTAG
GGATAACAGCATTATTTTTTATAAAGCTTATGACCTCGATGTTGGACTAGGGACTTTATGACTAGCCG
TCAAAATAGCATGTTCTGTTCGAACGTGACCCTACGTGATC
>Naranjia_sp_Vietnam_487
AAAACATAGCCCTAAGAAGATATTTAGGGTAAGCCCTGCCCGGTGACGGAAGTTTAACGGCCGCAGTA
CCTTGACTGTGCTAAGGTAGCGTAATCAGTTGGCTTTTAAATGGAGTCTGGTATGAACGGGTTTATGG
GGGAAAGCTGTCTCAGGCTTAGATTTTGAAATTATTAATAAGGTGAAAATACCTTTTGTCAAAAAAAA
GACGAGAAGACCCTTAGAGCTTTTAATAAATTGTAGTATATACAATACTTTTTTGTTGGGGCGACACG
GAGACATTGAACTCTCTCTAATTTAAATTACTTGCCGACTTTTTGTAAGTAAGAATAAGCTACCTTAG
GGATAACAGCATTATTTTTTATAAAGCTTATGACCTCGATGTTGGACTAGGGACTTTATGACTAGCCG
TCAAAATAGCATGTTCTGTTCGAACGTGACCCTACGTGATC
>Naranjia_sp_Vietnam_488
AAAACATAGCCCTAAGAAGATATTTAGGGTAAGCCCTGCCCGGTGACGGAAGTTTAACGGCCGCAGTA
CCTTGACTGTGCTAAGGTAGCGTAATCAGTTGGCTTTTAAATGGAGTCTGGTATGAACGGGTCTATGG
GGGAAAGCTGTCTCAGGCTTAGATTTTGAAATTATTAATAAGGTGAAAATACCTTTTGTTAAAAAAAA
GACGAGAAGACCCTTAGAGCTTTTAATAAATTGTAGTATACACAATACTTTTTTGTTGGGGCGACACG
GAGACATTGAACTCTCTCTAATTTAAATTACTTGCCGACTTTTTGTAAGTAAGAATAAGCTACCTTAG
GGATAACAGCATTATTTTTTATAAAGCTTATGACCTCGATGTTGGACTAGGGACTTTATGACTAGCCG
TCAAAATAGCATGTTCTGTTCGAACGTGACCCTACGTGATC
>Glacidorbis_hedleyi
AAAACATAGTCTACTGAATTTATATAGGATATTTTCTGCTCACTGATCTTTTTTAAAGAGCCGCAGTA
TTTTAACTGTGCTAAGGTAGCATAATCAATTGTCTATTAATTGTAGTCTAGAATGAATGG--------
--
AAAAAATGTAGTAATATTATCATTTTAATTTGCTCATTAGGTGCAAATACCTTCAAAAATATAATAGA
CGAGAAGACCCTAAGAACTTTTTAAA-----------------
ATGTTTTTGTTGGGGCGACATTGAAGCATTTAAACCTTCATATTTTTTCATATTTGCCGGGTCTTT-
CAAGTAAAATTAAGTTACCTTAGGGATAACAGCGTAATTTTTTCAAAAGTTTGCGACCTCGATGTTGG
ATTAGGAACTTATTAACTAACCGTAATTAAAGAGAGTTCTGTTCGAACTT-ATCCTACATGATC
>Maningrida_arnhemensis
AAAACATAGCCTTAAGAAGATATTTAAGGTAAGCCCTGCCCGGTGA-
AATTTTTTAACGGCCGCAGTACCCTGACTGTGCTAAGGTAGCGTAATCAGTTGGCTTTTAAATGGAGT
CGAGAATGAATGGGTAAATGGGAAAAAGCTGTCTCATTAATATATTTTGAATTTCCTAAGAAGATGAA
AATTTCTTCGATTAAAAAAAAGACGAGAAGACCCTTAGAGTTTTCATAAAATGA----
TTGACCATTACTTTTTTGTTGGGGCGACATAGAGACATCTAACTTTCTTAATAATTTGTGACAAGCCG
GCGCTTAGCAAGTAAGAATAAACTACCTTAGGGATAACAGCATTATTTTTTATAAAGCTTGTGACCTC
GATGTTGGACTAGGGACTAAGAGACTAGCCGTCAACTTAGCATGTTCTGTTCGAACAAAACCCTACAT
GATC
>Phallomedusa_austrina
AAAACATAGCCCTAAGAAATTATTTAGGGTAAGCCCTGCCCGGTGACGTGAGTTTAACGGCCGCAGTA
CCTTGACTGTGCTAAGGTAGCGTAATCAGTTGGCTTTTAAATGGAGTCTAGTATGAATGGGTTTATGG
GGGGTAGCTGTCTCAAGTTTGTGCTTTGAAATTCCTAAGAAGGTGAAAATTCCTTCAAGCAAATAAAA
GACGAGAAGACCCTTAGAGCTTTGAATAAAATATAGAATATAT-
TTACTTTTTTGTTGGGGCGACATGGAGACATTAAACTCTCCTTTGGAAAAGATACTTGCCGACGAATT
GTAAGTACGAATAAGCTACCTTAGGGATAACAGCATAATTTTCTTTTAAGCTTGTGACCTCGATGTTG
GACTAGGGACTTTATGACTAGCCGTCAAATTAGCATGTTCTGTTCGAACACGACCCTACATGATC
>P_solida
AAAACATAGCCCTAAGAAATTATTTAGGGTAAGCCCTGCCCGGTGACGTGAGTTTAACGGCCGCAGTA
CCTTGACTGTGCTAAGGTAGCGTAATCAGTTGGCTTTTAAATGGAGTCTAGTATGAATGGGTTTATGG
GGGGTAGCTGTCTCAAGTTTGTGCTTTGAAATTCCTAAGAAGGTGAAAATTCCTTCAAGCAAATAAAA

GACGAGAAGACCCTTAGAGCTTTGAATAAAATATAGAATATAT-
TTCCTTTTTTGTTGGGGCGACATGGAGACATTAAACTCTCCTTCGTAAAAGTTACTTGCCGACGAATT
GTAAGTACGAATAAGCTACCTTAGGGATAACAGCATAATTTTCTTTTAAGCTTGTGACCTCGATGTTG
GACTAGGGACTTTATGACTAGCCGTCAAATTAGCATGTTCTGTTCGAACACGACCCTACATGATC
>Amphibola_crenata
AAAACATAGCCCTAAGAAATGATTTAGGGTAAGCCCTGCCCAGTGATAACTGTTCAACGGCCGCAGTA
CCCTGACTGTGCTAAGGTAGCGTAATCAGTTGGCTTTTAATTGGAGTCGAGAATGAACGGGTCAATGG
GGAAAAGCTGTCTCATATTTGTATATTGAATTTACTAAGAAGATGAAAATTTCTTCAGAAAAACAAAA
GACGAGAAGACCCTTAGAGTTTTTAATAAATAAATGTTTGATTTATACTTTTTTGTTGGGGCGACATA
GAGACATTAAACTCTCTTTATTTTGTTTTACTTGCCGGCGTTTTGCAAGTGTGAATAAACTACCTTAG
GGATAACAGCATTATTTTTTATAAAGCTTGTGACCTCGATGTTGGACTAGGGACTAAATGACTAGCCG
TCAAATTAGCATGTTCTGTTCGAACAAAACCCTACATGATC
>Salinator_rosacea_1
AAAACATAGCCCTAAGAATATATTTAGGGTAAGCCCTGCCCGGTGATA---
GTTTAACGGCCGCAGTACCTTGACTGTGCTAAGGTAGCGTAATCAGTTGGCTTTTAAATGGAGTCTAG
TATGAATGGGTTCATGGGGGACAGCTGTCTCAAGCTTAGTGTTCGAATTTTCTAAGAAGGTGAAAATA
CCTTTAATAAACCAAAAGACGAGAAGACCCTTAGAGC-TTTAGTAAGTCGTA-
TATATACGATATCTTTTTGTTGGGGCGACATAGAGACAAATAACTCTCTTCAATTTAATTTACTTGCC
GACTTTTTGTAAGCAAGAATAAGCTACCTTAGGGATAACAGCATTATTTTTAGAAAAGCTTATGACCT
CGATGTTGGACTAGGGACTTTATGACTAGCCGTCAAAATAGCAGGCTCTGTTCGAGCATGACCCTACA
TGATC
>S_rosacea_2
AAAACATAGCCCTAAGAATATATTTAGGGTAAGCCCTGCCCGGTGATA---
GTTTAACGGCCGCAGTACCTTGACTGTGCTAAGGTAGCGTAATCAGTTGGCTTTTAAATGGAGTCTAG
TATGAATGGGTTCATGGGGGACAGCTGTCTCAAGCTTAGTGTTCGAATTTTCTAAGAAGGTGAAAATA
CCTTTAATAAACCAAAAGACGAGAAGACCCTTAGAGC-TTTAGTAAGTCGTA-
TATATACGATATCTTTTTGTTGGGGCGACATAGAGACAAATAACTCTCTTCAATTTAATTTACTTGCC
GACTTTTTGTAAGTAAGAATAAGCTACCTTAGGGATAACAGCATTATTTTTAAAAAAGCTTATGACCT
CGATGTTGGACTAGGGACTTTATGACTAGCCGTCAAAATAGCAAGCTCTGTTCGAGCATGACCCTACA
TGATC
>Lactiforis_tropicalis_1
AAAACATAGCCCTAAGAATATATTTAGGGTAAGCCCTGCCCGGTGATA--
AATTTAACGGCCGCAGTACCTTGACTGTGCTAAGGTAGCGTAATCAGTTGGCTTTTAAATGGAGTCTA
GTATGAATGGGTTTATGGGGAATAGCTGTCTCAAACTTAGAATTTGAATTTTTTAAGAAGGTGAAAAT
GCCTTCAACAAAATAAAAGACGAGAAGACCCTTAGAGCTTTTAGTAAGTTGTAACATGTACAATATCT
TTTTGTTGGGGCGACATGGAGACAAATAACTCTCCTAGGTGTTAATTACTTGCCGACTTTTTGTAAGT
AAGAATAAGCTACCTTAGGGATAACAGCATTATTTTTTA-
AAAGCTTATGACCTCGATGTTGGACTAGGGACTTTATGACTAGCCGTCAAAATAGCAGGCTCTGTTCG
AGCATGACCCTACATGATC
>L_tropicalis_2
AAAACATAGCCCTAAGAATATATTTAGGGTAAGCCCTGCCCGGTGATA--
AATTTAACGGCCGCAGTACCTTGACTGTGCTAAGGTAGCGTAATCAGTTGGCTTTTAAATGGAGTCTA
GTATGAATGGGTTTATGGGGAATAGCTGTCTCAAACTTAGAATTTGAATTTCTTAAGAAGGTGAAAAT
TCCTTCAACAAAATAAAAGACGAGAAGACCCTTAGAGCTTTTAGTAAGTTGTAACATGTACAATATCT
TTTTGTTGGGGCGACATGGAGACAAATAACTCTCCTAGATGTTAATTACTTGCCGACTTTTTGTAAGT
AAGAATAAGCTACCTTAGGGATAACAGCATTATTTTTTA-
AAAGCTTATGACCTCGATGTTGGACTAGGGACTTTATGACTAGCCGTCAAAATAGCAGGCTCTGTTCG
AGCATGACCCTACATGATC
>Lactiforis_sp_Johor
AAAACATAGCCCTAAGAATATATTTAGGGTAAGCCCTGCCCGGTGATAAATATTTAACGGCCGCAGTA
CCTTGACTGTGCTAAGGTAGCGTAATCAGTTGGCTTTTAAATGGAGTCTAGTATGAATGGGTTCATGG
GGGAAAGCTGTCTCAAACTTAGAATTCGAATTTCCTAAAAAGGTGAAAATACCTTTAATTAAATAAAA
GACGAGAAGACCCTTAGAGCTTTTAGTAAATTGTAGTATATACAATATCTTTTTGTTGGGGCGACATA
GACACAATTAACTCTCTTAAATGTTATTTACTTGCCGACTTTTTGTAAGTAAGAATAAGCTACCTTAG

GGATAACAGCATTATTTTTTAAAAAGCTTATGACCTCGATGTTGGACTAGGGACTTTATGACTAGCCG
TCAAATTAGCAAGCTC-------------------------
>L_takii_1
AAAACATAGCCCTAAGAATATATTTAGGGTAAGCCCTGCCCGGTGATAAGTATTTAACGGCCGCAGTA
CCTTGACTGTGCTAAGGTAGCGTAATCAGTTGGCTTTTAAATGGAGTCTAGTATGAATGGGTTTATGG
GGGAAAGCTGTCTCAAGCTTAGAAATCGAATTTTCTAAAAAGGTGAAAATGCCTTTAATTAAATAAAA
GACGAGAAGACCCTTAGAGCTTTTAGTAAATTGTAGTATATACAATATCTTTTTGTTGGGGCGACATA
GAGACAATTAACTCTCTAAAATGTTATTTACTTGCCGACTTTTTGTAAGTAAGAATAAGCTACCTTAG
GGATAACAGCATTATTTTTTAAAAAGCTTATGACCTCGATGTTGGACTAGGGACTTTATGACTAGCCG
TCAAATTAGCATGCTCTGTTCGAGCAAGACCCTACATGATC
>L_takii_2
AAAACATAGCCCTAAGAATATATTTAGGGTAAGCCCTGCCCGGTGATAAGTATTTAACGGCCGCAGTA
CCTTGACTGTGCTAAGGTAGCGTAATCAGTTGGCTTTTAAATGGAGTCTAGTATGAATGGGTTTATGG
GGGAAAGCTGTCTCAAGCTTAGAAATCGAATTTTCTAAAAAGGTGAAAATGCCTTTAATTAAATAAAA
GACGAGAAGACCCTTAGAGCTTTTAGTAAATTGTAGTATATACAATATCTTTTTGTTGGGGCGACATA
GAGACAATTAACTCTCTAAAATGTTATTTACTTGCCGACTTTTTGTAAGTAAGAATAAGCTACCTTAG
GGATAACAGCATTATTTTTTAAAAAGCTTATGACCTCGATGTTGGACTAGGGACTTTATGACTAGCCG
TCAAATTAGCATGCTCTGTTCGAGCAAGACCCTACATGATC
>S_fragilis_1
AAAACATAGCCCTAAGAACATATTTAGGGTGAGCCCTGCCCGGTGACGAAAGTTCAACGGCCGCAGTA
CCTTGACTGTGCTAAGGTAGCGTAATCAGTTGGCTTTTAAATGGAGTCTAGTATGAATGGGACCATGG
GGGAAAGCTGTCTCAAACTTATATTTTGAACTTATTATTAAGGTGAAAATACCTTTTTTTAATTAAAA
GACGAGAAGACCCTTAGAG-TTTTAATAAATTGTTTT-
TATACAATACTTTTTTGTTGGGGCGACGCGGAGACAAAAAACTCTCCTTCTTATACTTTACTTGCCGA
CTTCTAGTAAGTAAGAATAAACTACCTTAGGGATAACAGCATAATTTTTTATAAAGCTTATGACCTCG
ATGTTGGACTAGGGACTTTATGACTAGCCGTCAAAATAGCA---------------------------
---
>S_fragilis_2
AAAACATAGCCCTAAGAACATATTTAGGGTGAGCCCTGCCCGGTGACGAAAGTTCAACGGCCGCAGTA
CCTTGACTGTGCTAAGGTAGCGTAATCAGTTGGCTTTTAAATGGAGTCTAGTATGAATGGGACCATGG
GGGAAAGCTGTCTCAAACTTATATTTTGAACTTATTATTAAGGTGAAAATACCTTTTTTTAATTAAAA
GACGAGAAGACCCTTAGAG-
TTTTAATAAATTGTTCTTTATACAATACTTTTTTGTTGGGGCGACGCGGAGACAAAAAACTCTCCTTC
TTATACTTTACTTGCCGACTTCTAGTAAGTAAGAATAAACTACCTTAGGGATAACAGCATAATTTTTT
ATAAAGCTTATGACCTCGATGTTGGACTAGGGACTTTATGACTAGCCGTCAAAATAGCA---------
----
>S_rhamphidia
AAAACATAGCCCTAAGAAAATATTTAGGGTGAGCCCTGCCCGGTGACAAATTTTTAACGGCCGCAGTA
CCTTGACTGTGCTAAGGTAGCGTAATCAGTTGGCTTTTAAATGGAGTCTTGTATGAACGGGAACATGG
GGGAAAGCTGTCTCAAGTTTAGGAATCGAAGTTGCTAATAAGGTGAAAATGCCTTAACTAAATTAAAA
GACGAGAAGACCCTTAGAGC-
TTTAATAAATTGAATTATGTTCAATACTTTTTTGTTGGGGCGACAAGGAGACAACGAACTCTCCCTTT
TTTGTAGAACTTGCCGACTGATTGTAAGAAAGAATAAGCTACCTTAGGGATAACAGCATTATTTTTTA
TAAAGCTTATGACCTCGATGTTGGACTAGGGACTTTATGACTAGCCGTCAGATTAGCATGTTCTGTTC
GAACATGACCCTACATGATC
>S_tecta
AAAACATAGCCCTAAGAAGATATTTAGGGTGAGCCCTGCCCGGTGACATAAGTTTAACGGCCGCAGTA
CCTTGACTGTGCTAAGGTAGCGTAATCAGTTGGCTTTTAAATGGAGTCTAGTATGAATGGGAGCATGG
GGGGAAGCTGTCTCAAGCTTAGGTATCGAATTTATTAAAAAGGTGAAAATACCTTTATTTACCTAAAA
GACGAGAAGACCCTTAGAGC-
TTTAATAAATTGTATTTTGTACAATATTTTTTTGTTGGGGCGACATGGAGACAATAAACTCTCCTACA
TACACCTTACTTGCCGACTTATTGTAAGTAAGAATAAGCTACCTTAGGGATAACAGCATTATTTTTTA
TAAAGCTTATGACCTCGATGTTGGACTAGGGACTTTATGACTAGCCGTCAAAATAGCATGTTCTGTTC
GAACGTGACCCTACATGATC

>N_burmana_1
AAAACATAGCCCTAAGAATATATTTAGGGTGAGCCCTGCCCGGTGACGAAAGTTTAACGGCCGCAGTA
CCTTGACTGTGCTAAGGTAGCGTAATCAGTTGGCTTTTAAATGGAGTCTAGTATGAATGGGTTTATGG
GGGAAAGCTGTCTCAGGTTTAGAATTTGAATTTATTAAAAAGGTGAAAATACCTTTAATTAAAAAAAA
GACGAGAAGACCCTTAGAGCTTTTAATAAATTGTATAATGTATAATACTTTTTTGTTGGGGCGACATG
GAGACAAAAAACTCTCCTAATTTTAATTTACTTGCCGACTTTTTGTAAGTAAGAATAAGCTACCTTAG
GGATAACAGCATTATTTTTTATAAAGCTTATGACCTCGATGTTGGACTAGGGACTTTATGGCTAGCCG
CCAAAATAGCACGTTCTGTTCGAACATGACCCTACATGATC
>N_burmana_2
AAAACATAGCCCTAAGAATATATTTAGGGTGAGCCCTGCCCGGTGACGAAAGTTTAACGGCCGCAGTA
CCTTGACTGTGCTAAGGTAGCGTAATCAGTTGGCTTTTAAATGGAGTCTAGTATGAATGGGTTTATGG
GGGAAAGCTGTCTCAGGTTTAGAATTTGAATTTATTAAAAAGGTGAAAATACCTTTAATTAAAAAAAA
GACGAGAAGACCCTTAGAGCTTTTAATAAATTGTATAATGTATAATACTTTTTTGTTGGGGCGACATG
GAGACAAAAAACTCTCCTAATTTTAATTTACTTGCCGACTTTTTGTAAGTAAGAATAAGCTACCTTAG
GGATAACAGCATTATTTTTTATAAAGCTTATGACCTCGATGTTGGACTAGGGACTTTATGGCTAGCCG
CCAAAATAGCACGTTCTGTTCGAACATGACCCTACATGATC
>N_swatowensis_1

CATAGCCCTAAGAAAGTATTTAGGGTAAGCCCTGCCCGGTGACGGAAGTTTAACGGCCGCAGTACCTT
GACTGTGCTAAGGTAGCGTAATCAGTTGGCTTTTAAATGGAGTCTAGTATGAATGGGTCTATGGGGGA
AAGCTGTCTCAGGCTTAGATTTTGAAATTATTAATAAGGTGAAAATACCTTTTGTTAAAAAAAAGACG
AGAAGACCCTTAGAGCTTTTAATAAGTTGTAGTATATACAGTATTTTTTTGTTGGGGCGACATGGAGA
CATAGAACTCTCTTTAATTTAAATTACTTGCCGACTTTTTGTAAGTAAGAATAAGCTACCTTAGGGAT
AACAGCATTATTTTTTATAAAGCTTATGACCTCGATGTTGGACTAGGGACTTTATGACTAGCCGTCAA
AATAGCATGTTCTGTTCGAACGTGACCCTACATGATC
>N_swatowensis_2
AAAACATAGCCCTAAGAAGGTATTTAGGGTAAGCCCTGCCCGGTGACGGAAGTTTAACGGCCGCAGTA
CCTTGACTGTGCTAAGGTAGCGTAATCAGTTGGCTTTTAAATGGAGTCTAGTATGAATGGGTCTATGG
GGGAAAGCTGTCTCAGGCTTAGATTTTGAAATTATTAATAAGGTGAAAATACCTTTTGTTAAAAAAAA
GACGAGAAGACCCTTAGAGCTTTTAATAAGTTGTAGTATATACAATATTTTTTTGTTGGGGCGACATG
GAGACACAGAACTCTCTTTAATTTAAATTACTTGCCGACTTTTTGTAAGTAAGAATAAGCTACCTTAG
GGATAACAGCATTATTTTTTATAAAGCTTATGACCTCGATGTTGGACTAGGGACTTTATGACTAGCCG
TCAAAATAGCATGTTCTGTTCGAACGTGACCCTACATGA--
>Naranjia_sp_KhorZawrah_1
AAAACATAGCCCTAAGAACATATTTAGGGTGAGCCCTGCCCGGTGACGAAAGTTTAACGGCCGCAGTA
CCTTGACTGTGCTAAGGTAGCGTAATCAGTTGGCTTTTAAATGGAGTCTAGTATGAATGGGTTTATGG
GGGAGAGCTGTCTCAGGTTTAGAATTTGAATTTATTAAGAAGGTGAAAATACCTTTAATTAAAAAAAA
GACGAGAAGACCCTTAGAGCTTTTAAAAAATTGTATAATGTATAATACTTTTTTGTTGGGGCGACATG
GAGACAAAAAACTCTCCTTATTATGATTTACTTGCCGACCTTTTGTAAGTAAGAATAAGCTACCTTAG
GGATAACAGCATTATTTTTTATAAAGCTTATGACCTCGATGTTGGACTAGGGACTTTATGGCTAGCCG
CCAAAATAGCACGTTCTGTTCGAACATGACCCTACATGATC
>Naranjia_sp_KhorZawrah_2
AAAACATAGCCCTAAGAACATATTTAGGGTGAGCCCTGCCCGGTGACGAAAGTTTAACGGCCGCAGTA
CCTTGACTGTGCTAAGGTAGCGTAATCAGTTGGCTTTTAAATGGAGTCTAGTATGAATGGGTTTATGG
GGGAGAGCTGTCTCAGGTTTAGAATTTGAATTTATTAAGAAGGTGAAAATACCTTTAATTAAAAAAAA
GACGAGAAGACCCTTAGAGCTTTTAATAAATTGTATAATGTATAATACTTTTTTGTTGGGGCGACATG
GAGACAAAAAACTCTCCTTATTATGATTTACTTGCCGACCTTTTGTAAGTAAGAATAAGCTACCTTAG
GGATAACAGCATTATTTTTTATAAAGCTTATGACCTCGATGTTGGACTAGGGACTTTATGGCTAGCCG
CCAAAATAGCACGTTCTGTTCGAACATGACCCTACATGATC
>Naranjia_sp_Johor
AAAACATAGCCCTAAGAAGATATTTAGGGTAAGCCCTGCCCGGTGACGGAAGTTTAACGGCCGCAGTA
CCTTGACTGTGCTAAGGTAGCGTAATCAGTTGGCTTTTAAATGGAGTCTAGTATGAATGGGTCTATGG
GGGAAAGCTGTCTCAGGCTTAGATTTTGAAATTATTAATAAGGTGAAAATACCTTTTGTTAAAAAAAA
GACGAGAAGACCCTTAGAGCTTTTAATAAGTTGTAGTATATACAATATTTTTTTGTTGGGGCGACATG

```
GAGACATAAAACTCTCTTTAATTTAAATTACTTGCCGACTTTTTGTAAGTAAGAATAAGCTACCTTAG
GGATAACAGCATTATTTTTTATAAAGCTTATGACCTCGATGTTGGACTAGGGACTTTATGACTAGCCG
TCAAAATAGCATGTTCTGTTCGAACATGACCCTACATGATC
>Naranjia_sp_Singapore_1
AAAACATAGCCCTAAGAAGATATTTAGGGTAAGCCCTGCCCGGTGACGGAAGTTTAACGGCCGCAGTA
CCTTGACTGTGCTAAGGTAGCGTAATCAGTTGGCTTTTAAATGGAGTCTAGTATGAATGGGTCTATGG
GGGAAAGCTGTCTCAGGCTTAGATTTTGAAATTATTAATAAGGTGAAAATACCTTTTGTTAAAAAAAA
GACGAGAAGACCCTTAGAGCTTTTAATAAGTTGTAGTATATACAATATTTTTTTGTTGGGGCGACATG
GAGACATAAAACTCTCTTTAATTCAAATTACTTGCCGACT–
TTTGTAAGTAAGAATAAGCTACCTTAGGGATAACAGCATTATTTTTTATAAAGCTTATGACCTCTATG
TTGGACTAGGGACTTTATGACTAGCCGTCAAAATAGCATGTTCTGTTCGAACATGACCCTACATGATC
>Naranjia_sp_Singapore_2
AAAACATAGCCCTAAGAAGATATTTAGGGTAAGCCCTGCCCGGTGACGGAAGTTTAACGGCCGCAGTA
CCTTGACTGTGCTAAGGTAGCGTAATCAGTTGGCTTTTAAATGGAGTCTAGTATGAATGGGTCTATGG
GGGAAAGCTGTCTCAGGCTTAGATTTTGAAATTATTAATAAGGTGAAAATACCTTTTGTTAAAAAAAA
GACGAGAAGACCCTTAGAGCTTTTAATAAGTTGTAGTATATACAATATTTTTTTGTTGGGGCGACATG
GAGACATAAAACTCTCTTTAATTCAAATTACTTGCCGACTTTTTGTAAGTAAGAATAAGCTACCTTAG
GGATAACAGCATTATTTTTTATAAAGCTTATGACCTCGATGTTGGACTAGGGACTTTATGACTAGCCG
TCAAAATAGCATGTTCTGTTCGAACATGACCCTACATGATC
>Naranjia_sp_Bangkok_1
AAAACATAGCCCTAAGAAGATATTTAGGGTAAGCCCTGCCCGGTGACGGAAGTTTAACGGCCGCAGTA
CCTTGACTGTGCTAAGGTAGCGTAATCAGTTGGCTTTTAAATGGAGTCTGGTATGAACGGGTCTATGG
GGGAAAGCTGTCTCAGGCTTAGATTTTGAAATTATTAATAAGGTGAAAATACCTTTTGTTAAAAAAAA
GACGAGAAGACCCTTAGAGCTTTTAATAAATTGTAGTATATACAATACTTTTTTGTTGGGGCGACACG
GAGACATTGAACTTTCTCTAATTTAAATTACTTGCCGACTTTTTGTAAGTAAGAATAAGCTACCTTAG
GGATAACAGCATTATTTTTTATAAAGCTTATGACCTCGATGTTGGACTAGGGACTTTATGACTAGCCG
TCAAAATAGCATGTTCTGTTCGAACGTGACCCTACATGATC
>Naranjia_sp_Bangkok_2
AAAACATAGCCCTAAGAAGATATTTAGGGTAAGCCCTGCCCGGTGACGGAAGTTTAACGGCCGCAGTA
CCTTGACTGTGCTAAGGTAGCGTAATCAGTTGGCTTTTAAATGGAGTCTGGTATGAACGGGTCTATGG
GGGAAAGCTGTCTCAGGCTTAGATTTTGAAATTATTAATAAGGTGAAAATACCTTTTGTTAAAAAAAA
GACGAGAAGACCCTTAGAGCTTTTAATAAATTGTAGTATACACAATACTTTTTTGTTGGGGCGACACG
GAGACATTGAACTCTCTCTAATTTAAATTACTTGCCGACTTTTTGTAAGTAAGAATAAGCTACCTTAG
GGATAACAGCATTATTTTTTATAAAGCTTATGACCTCGATGTTGGACTAGGGACTTTATGACTAGCCG
TCAAAATAGCATGTTCTGTTCGAACGTGACCCTACATGATC
```

Supplementary Information 3

```
>Naranjia_sp_Vietnam_486
-----------------------------------------
ATCTCCCCAGTAACGGCGAGTGAAGCGGGAAGAGCCCAGCACCGAATCCCCCGGCGCGCCGCCGGCGG
GAACTGTGGTGTGCGGGACGCCACCAGTCGCGCGTGCCGGCGCCCAAGTCCTCCTGATCGAGGCTTCA
CCCGGAGCGGGTGTAAGGCCTTTGGCAGGTGCCCGGCTGCGCGGCCGCGAGCGTCCCAGGAGTCGGGT
TGTTTGGGAATGCAGCCCAAAGCGGGTGGTAAACTCCATCTAAGGCTAAATACCGGCACGAGTCCGAT
AGCGGACAAGTACCGTGAGGGAAAGTTGAAAAGAACTTTGAAGAGAGAGTTCAAGAGTACGTGAAACC
GCCCAGAGGTAAACGGGTGGATCCGCGAAGTCGGCCCGCGAAATTCAGCGCGGCGGTCGGGCGCGCGC
GCCGGGAGTGGGGGATCCTTCGAGGACCCCGCCCGGCGCGGCTCGGCGTCGCACCGCCGCGTGCACTT
TTCGCGGGCGGAGCGCCACGACCGGTTCCTCGGCGGACACACGCCGGCGGCGGGAAGGTAGGTGCGCC
GCCCGTCGGCGCACTGTTACAGCCCGCCCCGGAGGCGCCCGCCGAAGGGACCGAGGAACGGCCGCCAG
CCCTCGAGGCCGCCCGGCCTCCCCGGGGGAGTCCGACCGGCGGGGACTGGGTTCAAGCCCGTGCCCGC
CGACCGCTCCCCCGCGCAG----
CCGGGCGTCGGCCGGGGCCGGCTCGGGGTCGGTGGCGAATCTGTCGGCATTCCACCCGACCCGTCTTG
AAACACGGACCAAGGAGTCTAACATGCGCGCGAGTCATTGGGCCGTACGAAACCCAAAGGCGCAGTGA
AAGCGAGGGCCGTCTCGGACCGGCCCAGGTGGGATCCCCTCGACCTCCTCGGAGGAGGGGGGCGCACC
ACCGGCCCGTCCCGTCCGCGCCGTCGGTGGGGCGGAGCAGGAGCGTGCACGCTGGGACCCGAAAGATG
GTGAACTATGCCTGAGTAGGACGAAGCCAGA-----------------------------------
>Naranjia_sp_Vietnam_487
-----------------------------------------
ATCCCCCCAGTAACGGCGAGTGAAGCGGGAAGAGCCCAGCACCGAATCCCCCGGCGCGCCGCCGGCGG
GAACTGTGGTGTGCGGGACGCCACCAGTCGCGCGTGCCGGCGCCCAAGTCCTCCTGATCGAGGCTTCA
CCCGGAGCGGGTGTAAGGCCTTTGGCAGGTGCCCGGCTGCGCGGCCGCGAGCGTCCCAGGAGTCGGGT
TGTTTGGGAATGCAGCCCAAAGCGGGTGGTAAACTCCATCTAAGGCTAAATACCGGCACGAGTCCGAT
AGCGGACAAGTACCGTGAGGGAAAGTTGAAAAGAACTTTGAAGAGAGAGTTCAAGAGTACGTGAAACC
GCCCAGAGGTAAACGGGTGGATCCGCGAAGTCGGCCCGCGAAATTCAGCGCGGCGGTCGGGCGCGCGC
GCCGGGAGTGGGGGATCCTTCGAGGACCCCGCCCGGCGCGGCTCGGCGTCGCACCGCCGCGTGCACTT
TTCGCGGGCGGAGCGCCACGACCGGTTCCTCGGCGGACACACGCCGGCGGCGGGAAGGTAGGTGCGCC
GCCCGTCGGCGCACTGTTACAGCCCGCCCCGGAGGCGCCCGCCGAAGGGACCGAGGAACGGCCGCCAG
CCCTCGAGGCCGCCCGGCCTCCCCGGGGGAGTCCGACCGGCGGGGACTGGGTTCAAGCCCGTGCCCGC
CGACCGCTCCCCCGCGCAG----
CCGGGCGTCGGCCGGGGCCGGCTCGGGGTCGGTGGCGAATCTGTCGGCATTCCACCCGACCCGTCTTG
AAACACGGACCAAGGAGTCTAACATGCGCGCGAGTCATTGGGCCGTACGAAACCCAAAGGCGCAGTGA
AAGCGAGGGCCGTCTCGGACCGGCCCAGGTGGGATCCCCTCGACCTCCTCGGAGGAGGGGGGCGCACC
ACCGGCCCGTCCCGTCCGCGCCGTCGGTGGGGCGGAGCAGGAGCGTGCACGCTGGGACCCGAAAGATG
GTGAACTATGCCTGAGTAGGACGAAGCCAGA-----------------------------------
>Glacidorbis_hedleyi
TTAAGCATATTACTAAGCGGAGGAAAAGAAACTAACAAGGATTTCCCCAGTAACGGCGAGTGAAGCGG
GAAGAGCCCAGCACCGAATCCCCCGGTGTCATGCCGGCGGGAACTGTGGTGTGTGGGACGCCACCAGT
CGCACATGCGGGCACCAAAGTCCTCCTGATCGAGGCTTTACCCAGAGTGGGTGTAAGGCCTTT-
GCCGGTGCCTCGCTGTGCGGCCGCGAGCGTCTCAGGAGTCGGGTTGTTTGGGAATGCAGCCCAAAGCG
GGTGGTAAACTCCATCTAAGGCTAAATACTGGCACGAGTCCGATAGCGGACAAGTACCGTGAGGGAAA
GTTGAAAAGAACTTTGAAGAGAGAGTTCAAGAGTACGTGAAACCGCACACAGGTAAACGGGTGGATCC
GCAAAGTCGGCCCGCGGAATTCAGCGCGGCGAGCGGCCCGGGGCGCCTGGGTTC-GGGATCCTCT-
GTGACCCGCCCGGGTGCCGCGCTGGGCC---
CCGCCGCGTGCACTTTCCGCGGGCAGAGCGCCACGACCGGTTTCTCGCCGGCCATAAGCCGG--
GTGGGAAGGTAGG-----------CCTGCGGGCTGTTATAGACCACCACGGTA--GGCCGGTG-
CGGGACCGAGGGACGACCGC--GCCTTCGAGGCTACCCGGCCT-
TCCGGGGGAGTTCGACTGGCAGAGACTGGGC----
AACCGTGTCTGCCGACCGCTCCTCCGCGCGGTTTTTC--
GGGCTAGCCGGGACCTGCTGAGGGTCAGTGGCGAATCTGTCGGCATTCCACCCGACCCGTCTTGAAAC
ACGGACCAAGGAGTCTAACATGCGCGCGAGTCATTGGGTCGTACGAAACCCAAAGGCGCAGTGAAAGC
GAGGGTCGTCTCGGGCTGACCCAGGTGGGATCTCTGCTCCCCCTCGTG-
GGGGTGGAGCGCACCACCGGCCCGTCCCGTCTGCGTCGTCAGTGGGGCGGAGCAAGAGCGTGCACGCT
```

GGGACCCGAAAGATGGTGAACTATGCCTGAGTAGAACGAAGCCAGAGGAAACTCTGGTGGAAGTCCGT
AGCGATTCTGAC
>Maningrida_arnhemensis
-
TAAGCATATAACTAAGCGGAGGAAAAGAAACTAACAAGGATTTCCCCAGTAACGGCGAGTGAAGCGGG
AAGAGCCCAGCACCGAATCCCCGCTGTGACGGCGGCGGGAACTGTGGTGTGTGGGACGCCACCAGTC
GCGCGTGCGAGCGCCCAAGTCCTCCTGATCGAGGCTTCACCCAGAGCGGGTGTAAGGCCTTTGACGGT
GCCTTCGCTGCGCGGCCGCGAGCGTCCTAGGAGTCGGGTTGTTTGGGAATGCAGCCCAAAGCGGGTGG
TAAACTCCATCTAAGGCTAAATACTGGCACGAGTCCGATAGCGGACAAGTACCGTGAGGGAAAGTTGA
AAAGAACTTTGAAGAGAGAGTTCAAGAGTACGTGAAACCGCCCAGAGGTAAACGGGTGGATCCGCAAA
GTCGGCCCGCGGCATTCAGCGCGGCGCGCAGCGGGGGGCGCTCGGGCTC-GGGATCCCTG--
GGACCCGCTCGGGCGCCG---
CGCCCCGCTCCGCCGCGTGCACTTGCCGCGGGCAGAGCGCCACGACCGGTTTCGCGGCGGCCATAAAC
CGACGGCGGGAAGGTAGGCGC------
CGGCGGCGCGCTGTTATAGACCGCCTCGGAAGCGCCCGCCG-
CGGGACCGAGGGACGGCCGCGCGACCTCGAGGCCGCCCGGCCAACCCGGGGGGGTTCGACTGGCAGGG
ACTGGGC----GACCGTGCCTGCCGACCGCCTCCCCGGACGGA---
CCCGGGCCGGCCCGGTGTTCGCTGAGGGTCGGTGGCGAATCTGTCGGCATTCCACCCGACCCGTCTTG
AAACACGGACCAAGGAGTCTAACATGCGCGCGAGTCATTGGGCTGTACGAAACCCAAAGGCGCAGTGA
AAGCGAGGGTCGTCTCGGGCTGACCCAGGTGGGATCCCTTC---CTCCGCGGAG--
GGGGGGCGCACCACCGGCCCGTCCCGTCTGCGTCGTCAGTGGGGCGGAGCAAGAGCGTGCACGCTGGG
ACCCGAAAGATGGTGAACTATGCCTGAGTAGAACGAAGCCAGAGGAAACTCTGGTGGAAGTCCGTAGC
GATTCTGAC
>Phallomedusa_austrina
TTAAGCATATAACTAAGCGGAGGAAAAGAAACTAACAAGGATTTCCCCAGTAACGGCGAGTGAAGCGG
GAAGAGCCCAGCACCGAATCCCCGGTGTTCAGCCGGCGGGAACTGTGGTGTGTGGGACGCCACCAGT
CGCGCATGCCGATGCCCAAGTCCTCCTGATCGAGGCTTCACCCAGAGCGGGTGTAAGGCCTTTAGCTG
GTACTCGGCTGCGCGGCCGCGAGCGTCCCAGGAGTCGGGTTGTTTGGGAATGCAGCCCAAAGCGGGTG
GTAAACTCCATCTAAGGCTAAATACCGGCACGAGTCCGATAGCGGACAAGTACCGTGAGGGAAAGTTG
AAAAGAACTTTGAAGAGAGAGTTCAAGAGTACGTGAAACCGCTCAGAGGTAAACGGGTGGATCCGCAA
AGTCGGCCCGCGAGATTCAGCGCGGCGGAC-
GGTGCGGGCGCCTGGCACCGGGGATCCTCTGCGGACCCGGCCAGGCGCCG-
TCTGCGCCGCTCCGCCGCGTGCACTTCTCGCGGGCAGAGCGCCACGACCGGTTCCTCGGCGGCCACAA
GCCGGTGGCGGGAAGGTAGGTGCGTCGCCTGGCGGCGCACTGTTACAGCCCGTCTCGGAAGCGCCCGC
CGATGGGACCGAGGGACGGCCGCCTGCCCCCGAGGCCGCCCGGCCTCCCCGGGGGAGTTCGACTGGCA
GGGACTGGGTACCC-CCCGTGCCTGCCGACCGCTCCTCCGGACGGACATCC--
GGGTCGGCCGGGGCTCGCTCAGGGTCGGTGGCGAATCTGTCGGCATTCCACCCGACCCGTCTTGAAAC
ACGGACCAAGGAGTCTAACATGCGCGCGAGTCATTGGGCTGTACGAAACCCAAAGGCGCAGTGAAAGC
GAGGGCCGTCTCGGACTGGCCCAGGTGGGATCCCCTCCGGCCTCCCGG-----
GGGGGCGCACCACCGGCCCGTCCCGTCTGCGTCGTCAGTGGGGCGGAGCAGGAGCGTGCACGCTGGGA
CCCGAAAGATGGTGAACTATGCCTGAGTAGAACGAAGCCAGAGGAAACTCTGGTGGAAGTCCGTAGCG
ATTCTGAC
>P_solida
TTAAGCATATAACTAAGCGGAGGAAAAGAAACTAACAAGGATTTCCCCAGTAACGGCGAGTGAAGCGG
GAAGAGCCCAGCACCGAATCCCCGGTGTTCAGCCGGCGGGAACTGTGGTGTGTGGGACGCCACCAGT
CGCGCATGCCGATGCCCAAGTCCTCCTGATCGAGGCTTCACCCAGAGCGGGTGTAAGGCCTTTAGCTG
GTACTCGGCTGCGCGGCCGCGAGCGTCCCAGGAGTCGGGTTGTTTGGGAATGCAGCCCAAAGCGGGTG
GTAAACTCCATCTAAGGCTAAATACCGGCACGAGTCCGATAGCGGACAAGTACCGTGAGGGAAAGTTG
AAAAGAACTTTGAAGAGAGAGTTCAAGAGTACGTGAAACCGCTCAGAGGTAAACGGGTGGATCCGCAA
AGTCGGCCCGCGAGATTCAGCGCGGCGGAC-
GGTGCGGGCGCTTGGCACTGGGGATCCTCTGCGGACCCGGCCAGGCGCCG-
TCTGCGCCGTTCCGCCGCGTGCACTTCTCGCGGGCAGAGCGCCACGACCGGTTCCTCGGCGGCCACAA
ACCGGTGGCGGGAAGGTAGGTGCGTCGCCTGGCGGCGCACTGTTACAGCCCGTCCCGGAAGCGCCCGC
CGATGGGACCGAGGGACGGCCGCTTGCCCCCGAGGCCGCCCGGCCTCCCCGGGGGAGTTCGACTGGCA

GGGACTGGGTACCC-CCCGTGCCTGCCGACCGCTCCTCCGGACGGACATCC--
GGGTCGGCCGGGGCTCGCTCAGGGTCGGTGGCGAATCTGTCGGCATTCCACCCGACCCGTCTTGAAAC
ACGGACCAAGGAGTTTAACATGCGCGCGAGTCATTGGGCTGTACGAAACCCAAAGGCGCAGTGAAAGC
GAGGGCCGTCTCGGACTGGCCCAGGTGGGATCCCCTCCCCCCCTCGGGGTGCGGGGGGCGCACCACCG
GCCCGTCCCGTCTGCGTCGTCAGTGGGGCGGAGCAGGAGCGTGCACGCTGGGACCCGAAAGATGGTGA
ACTATGCCTGAGTAGAACGAAGCCAGAGGAAACTCTGGTGGAAGTCCGTAGCGATTCTGAC
>Amphibola_crenata
TTAAGCATATAACTAAGCGGAGGAAAAGAAACTAACAAGGATTTCCCCAGTAACGGCGAGTGAAGCGG
GAAGAGCCCAGCACCGAATCCCCCGGTGTGCAGCCGGCGGGAACTGTGGTGTGTGGGACGCCACCAGT
CGCGCATGCCGATGCCCAAGTCCTCCTGATCGAGGCTTCACCCAGAGCGGGTGTAAGGCCTTTAGCTG
GTACTCGGCTGCGCGGCCGCGAGCGTCCCAGGAGTCGGGTTGTTTGGGAATGCAGCCCAAAGCGGGTG
GTAAACTCCATCTAAGGCTAAATACCGGCACGAGTCCGATAGCGGACAAGTACCGTGAGGGAAAGTTG
AAAAGAACTTTGAAGAGAGAGTTCAAGAGTACGTGAAACCGCTCAGAGGTAAACGGGTGGATCCGCAA
AGTCGGCCCGCGAAATTCAGCGCGGCGGGC-
GGTGCGGGTGCCGGGTCCCTGGGATGCTCTGCGCACCCGGCCCGGCGCCC-
CCGACGCCGCTCCGCCGCGTGCACTTTTCGCGGGCAGAGCGCCACGACCGGTTCCCCGGCGGCCACAA
ACCGGCGGCGGGAAGGTAGGTGAGCTGCTCGGCAGCGCACTGTTACAGCCCGCCCCGGAGGCGCCCGC
CGA-
GGGACCGAGGGACGGCCGCTTGCCCTCGAGGCCGCCCGGCCTCCCCGGGGGAGTTCGACTGGCAGGGA
CTGGGTTCCC-CCCGTGCCTGCCGACCGCTCCTCCGGACGGAC-TCC--
GGGTCGGCCGGGGCTCGCTCAGGGTCGGTGGCGAATCTGTCGGCATTCCACCCGACCCGTCTTGAAAC
ACGGACCAAGGAGTCTAACATGCGCGCGAGTCATTGGGCTGTACGAAACCCAAAGGCGCAGTGAAAGC
GAGGGTCGTCTCGGACTGACCCAGGTGGGATCCCTCTCTTCACGCCCGCGGCGGGGGGCGCACCACCG
GCCCGTCCCGTCTGCGTCGTCAGTGGGGCGGAGCTAGAGCGTGCACGCTGGGACCCGAAAGATGGTGA
ACTATGCCTGAGTAGAACGAAGCCA-----------------------------------
>Salinator_rosacea_1
TTAAGCATATAACTAAGCGGAGGAAAAGAAACTAACAAGGATCCCCCCAGTAACGGCGAGTGAAGCGG
GAAGAGCCCAGCACCGAATCCCCCGGTGTGCAGCCGGCGGGAACTGTGGTGTGTGGGACGCCACCAGT
CGCGCATGCCGATGCCCAAGTCCTCCTGATCGAGGCTTCACCCAGAGCGGGTGTAAGGCCTTTAGCAG
GTACTCGGCTGCGCGGCCGCGAGCGTCTCAGGAGTCGGGTTGTTTGGGAATGCAGCCCAAAGCGGGTG
GTAAACTCCATCTAAGGCTAAATACCGGCACGAGTCCGATAGCGGACAAGTACCGTGAGGGAAAGTTG
AAAAGAACTTTGAAGAGAGAGTTCAAGAGTACGTGAAACCGCCCAGAGGTAAACGGGTGGATCCGCAA
AGTCGGCTCGCGAAATTCAGCGCGGCGGTCGGGCGCTGGCGTCCGGTCACAGGGATCCTCTGAGGACC
CGGCCGGGCGTCGTTCGGCGTCGCACCGCCGCGTGCACTTTTCGCGAGCAGAGCGCCACGACCGGTTC
CTCGGCGGCCACACACCGACGGCGGGAAGGTAGGTGCGTCGCTCGTCGGCGCACTGTTACAGCCCGTC
TCGGAGGCGCCCGCCGATGGGACCGAGGAACGGCCGCTTGCCCTCGAGGCCGCCCGGCCTCCCCGGGG
GAGTTCGACTGGTGGGGACTGGGTTCAAGCCCGTGCTCGCCGACCGCTCCTCCGGACGGACATTCCGG
GCTCGGCCGGGGCTAGCTCGGGGTCGGTGGCGAATCTGTCGGCATTCCACCCGACCCGTCTTGAAACA
CGGACCAAGGAGTCTAACATGCGCGCGAGTCATTGGGCTGTACGAAACCCAAAGGCGCAGTGAAAGCG
AGGGCCGTCTCGGACTGGCCCAGGTGGGATCCCTCT--
CTTCCCTCGGTGGCGGGGGCGCACCACCGGCCCGTCCCGTCCGCGTCGGCGGTGGGGCGGAGCAGGAG
CGTGCACGCTGGGACCCGAAAGATGGTGAACTATGCCTGAGTAGAACGAAGCCAGAG-----------
----
>Lactiforis_tropicalis_1
TTAAGCATATAACTAAGCGGAGGAAAAGAAACTAACAAGGATCCCCCCAGTAACGGCGAGTGAAGCGG
GAAGAGCCCAGCACCGAATCCCCCGGTGTGCAGCCGGCGGGAACTGTGGTGTGTGGGACGCCACCAGT
CGCGCATGCCGATGCCCAAGTCCTCCTGATCGAGGCTTCACCCAGAGCGGGTGTAAGGCCTTTAGCAG
GTACTCGGCTGCGCGGCCGCGAGCGTCTCAGGAGTCGGGTTGTTTGGGAATGCAGCCCAAAGCGGGTG
GTAAACTCCATCTAAGGCTAAACACCGGCACGAGTCCGATAGCGGACAAGTACCGTGAGGGAAAGTTG
AAAAGAACTTTGAAGAGAGAGTTCAAGAGTACGTGAAACCGCCCAGAGGTAAACGGGTGGATCCGCAA
AGTCGGCCCGCGAGATTCAGCGCGGCGGTCGGGCGCGAGCGTCGGGTCCCGGGGATCCTCTGAGGACC
CCGCCCGGCGTCG-
TCGGCGTCGCACCGCCGCGTGCACTTCTCGCGGGCAGAGCGCCACGACCGGTTCCTCGGCGGCCACAC
ACCGGCGGCGGGAAGGTAGGCGCGCCGCTCGTCGGCGCGCTGTTACAGCCCGTCCCGGAGGCGCCCGC

CGATGGGACCGAGGATCGGCCGCTCGCCCTCGAGGCCGCCCGGCCTCCCCGGGGGAGTTCGACTGGCG
GGGACTGGGTCAAAGCCCGTGCCCGCCGACCGCTCCCCTGGACGGAAATCC--
GGGTCGGCCGGGGCTCGCTCGGGGTCGGTGGCGAATCTGTCGGCACTCCACCCGACCCGTCCTGAAAC
ACGGACCAAGGAGTCTAACATGCGCGCGAGTCATTGGGCTGTACGAAACCCAAAGGCGCAGTGAAAGC
GAGGGCCGTCTCGGACTGGCCCAGGTGGGATCCCCTCTCCCTCTCGCGGGGGGGGGGCGCACCACCG
GCCCGTCCCGTCCGCGTCKGCGGTGGGGCGGAGCAGGAGCGTGCACGCTGGGACCCGAAAGATGGTGA
ACTATGCCTGAGTAGAACGAAACCAGAGGAAACTCTGGTGGAAGTCCGTAGCGATTCTGAC
>Lactiforis_sp_Johor
TTAAGCATATAACTAAGCGGAGGAAAAGAAACTAACAAGGATCCCCCCAGTAACGGCGAGTGAAGCGG
GAAGAGCCCAGCACCGAATCCCCCGGTGTGCAGCCGGCGGGAACTGTGGTGTGTGGGACGCCACCAGT
CGCGCATGCCGATGCCCAAGTCCTCCTGATCGAGGCTTCACCCAGAGCGGGTGTAAGGCCTTTAGCAG
GTACTCGGCTGCGCGGCCGCGAGCGTCTCAGGAGTCGGGTTGTTTGGGAATGCAGCCCAAAGCGGGTG
GTAAACTCCATCTAAGGCTAAATACCGGCACGAGTCCGATAGCGGACAAGTACCGTGAGGGAAAGTTG
AAAAGAACTTTGAAGAGAGAGTTCAAGAGTACGTGAAACCGCCCAGAGGTAAACGGGTGGATCCGCAA
AGTCGGCCCGCGAAATTCAGCGCGGCGGTCTGGCGCGGGCGTCGGGTCTC-
GGGATCCTCTGAGGACCCGGCCCGGCGTCGTTCGGCGTCGCACCGCCGCGTGCACTTTTCGCGGGCAG
AGCGCCACGACCGGTTCCTCGGCGGCCACACACCGGTGGCGGGAAGGTAGGTGCGCCGCTCGTCGGTG
CACTGTTACAGCCCGTCCCGGAGGCGCCCGCCGACGGGACCGAGGAACGGCCGCTTGCCCTCGAGGCC
GCCCGGCCTCCCCGGGGGAGTTCGACTGGCGGGGACTGGGTTCAAGCCCGTGCCCGCCGACCGCTCCT
CTGGACGGAAATCC--
GGGTCGGCCGGGGCTCGCTCGGGGTCGGTGGCGAATCTGTCGGCATTCCACCCGACCCGTCTTGAAAC
ACGGACCAAGGAGTCTAACATGCGCGCGAGTCATTGGGCTGTACGAAACCCAAAGGCGCAGTGAAAGC
GAGGGCCGTCTCGGACTGGCCCAGGTGGGATCCCTCC-
TCCGCTCGCGCRGAGGGGGGCGCACCCCCGGCCCGTCCCGTCCGCGTCCGCGGTGGGGCAGAACAAGA
GCGTGCACTCTGGGACCCGAAAGATGGTGAACTATGCCTGAATAAAACAAACCCAGAAGAAACTCTGG
TGGAAGTCCGTAGCGATTCTGAC
>S_fragilis_1
-
TAAGCATATAACTAAGCGGAGGAAAAGAAACTAACAAGGATCCCCCCAGTAACGGCGAGTGAAGCGGG
AATAGCCCAGCACCGAATCCCCCGGCGTGCCGCCGGCGGGAACTGTGGTGTGTGGGACGCCACCAGTC
GCGCATGCCGATGCCCAAGTCCTCCTGATCGAGGCTTCACCCAGAGCGGGTGTAAGGCCTTTAGCAGG
TACTCGGCTGCGCGGCCGCGAGCGTCTCAGGAGTCGGGTTGTTTGGGAATGCAGCCCAAAGCGGGTGG
TAAACTCCATCTAAGGCTAAATACCGGCACGAGTCCGATAGCGGACAAGTACCGTGAGGGAAAGTTGA
AAAGAACTTTGAAGAGAGAGTTCAAGAGTACGTGAAACCGCTCAGAGGTAAACGGGTGGATCCGCAAA
GTCGGCCCGCGAGATTCAGCGCGGCGGCCGGGCGCGCGCGTCGGGACGAGAGGACTCTCCGAGGACCC
CTCCCGGCGCGGTCGAGCGTCGCGCCGCCGCGTGCACTTTTCGCGGGCGGAGCGCCACGACCGGTTCC
TCGGCGGCCACACACCGGCGGCGGGAAGGTAGGCGCGTCTGTCACAGGCGCGCTGTTACAGCCCGTCC
CGGAGGCGCCCGCCGATGGGACCGAGGAACGGCCGCTTGCCCTCGAGGCCGCCCGGCCTCCCCGGGGG
AGTTCGACTGGCGGGGACTGGGTTCAAGCCCGTGCCCGCCGACCGCTCCTCCGGACGGAAATTCCGGG
CCCGGCCGGGGCTCGCTCGGGGTCGGTGGCGAATCTGTCGGCATTCCACCCGACCCGTCTTGAAACAC
GGACCAAGGAGTCTAACATGCGCGCGAGTCATTGGGCTGTACGAAACCCAAAGGCGCAGTGAAAGCGA
GGGCCGTCTCGGACTGGCCCAGGTGGGATCCCTCCTCCCCGCTCGCGGGAGGGGGGCGCACCACCGGC
CCGTCCCGTCTGCGTCGTCAGTGGGGCGGAGCAGGAGCGTGCACGCTGGGACCCGAAAGATGGTGAAC
TATGCCTGAGTAGAACGAAGCCAGAGGAAACTCTGGTGGAAGTCCGTAGCGATTCTGAC
>S_fragilis_2
TTAAGCATATAACTAAGCGGAGGAAAAGAAACTAACAAGGATCCCCCCAGTAACGGCGAGTGAAGCGG
GAATAGCCCAGCACCGAATCCCCCGGCGTGCCGCCGGCGGGAACTGTGGTGTGTGGGACGCCACCAGT
CGCGCATGCCGATGCCCAAGTCCTCCTGATCGAGGCTTCACCCAGAGCGGGTGTAAGGCCTTTAGCAG
GTACTCGGCTGCGCGGCCGCGAGCGTCTCAGGAGTCGGGTTGTTTGGGAATGCAGCCCAAAGCGGGTG
GTAAACTCCATCTAAGGCTAAATACCGGCACGAGTCCGATAGCGGACAAGTACCGTGAGGGAAAGTTG
AAAAGAACTTTGAAGAGAGAGTTCAAGAGTACGTGAAACCGCTCAGAGGTAAACGGGTGGATCCGCAA
AGTCGGCCCGCGAGATTCAGCGCGGCGGCCGGGCGCGCGCGTCGGGACGAGAGGACTCTCCGAGGACC
CCTCCCGGCGCGGTCGAGCGTCGCGCCGCCGCGTGCACTTTTCGCGGGCGGAGCGCCACGACCGGTTC
CTCGGCGGCCACACACCGGCGGCGGGAAGGTAGGCGCGTCTGTCACAGGCGCGCTGTTACAGCCCGTC

CCGGAGGCGCCCGCCGATGGGACCGAGGAACGGCCGCTTGCCCTCGAGGCCGCCCGGCCTCCCCGGGG
GAGTTCGACAGGCGGGGACTGGGTTCAAGCCCGTGCCCGCCGACCGCTCCTCCGGACGGAAATTCCGG
GCCCGGCCGGGGCTCGCTCGGGGTCGGTGGCGAATCTGTCGGCATTCCACCCGACCCGTCTTGAAACA
CGGACCAAGGAGTCTAACATGCGCGCGAGTCATTGGGCYGTACGAAACCCAAAGGCGCAGTGAAAGCG
AGGGCCGTCTCGGACTGGCCCAGGTGGGATCCCTCCTCCCCCGCTCGCGGGGGGGGGCGCACCACCGG
CCCGTCCCGTCTGCGTCGTCAGTGGGGCGGAGCAGGAGCGTGCACGCTGGGACCCGAAAGATGGTGAA
CTATGCCTGAGTAGAACGAAGCCAGAGGAAA------------------------------
>S_rhamphidia
TTAAGCATATAACTAAGCGGAGGAAAAGAAACTAACAAGGATCCCCCCAGTAACGGCGAGTGAAGCGG
GAAGAGCCCAGCACCGAATCCCCCGGCGTGCCGTCGGCGGGAACTGTGGTGTGTGGGACGCCACCAGT
CGCGCATGCCGATGCCCAAGTCCTCCTGATCGAGGCTTCACCCAGAGCGGGTGTAAGGCCTTTAGCAG
GTACTCGGCTGCGCGGCCGCGAGCGTCTCAGGAGTCGGGTTGTTTGGGAATGCAGCCCAAAGCGGGTG
GTAAACTCCATCTAAGGCTAAATACCGGCACGAGTCCGATAGCGGACAAGTACCGTGAGGGAAAGTTG
AAAAGAACTTTGAAGAGAGAGTTCAAGAGTACGTGAAACCGCTCAGAGGTAAACGGGTGGATCCGCAA
AGTCGGCCCGCGAGATTCAGCGCGGCGGCCGGGCGCGCGCGTCGGGACGAGAGGACTCTCTGAGGACC
CCTCCCGGCGCGGTCGAGCGTCGCGTCGCCGCGTGCACTTTTCGCGGGCAGAGCGCCACGACCGGTTC
CCCGGCGGCCACAAACCGGTGGCGGGAAGGTAGGTGCGCCGCTCGCCGGTGCACTGTTACAGCCCGTC
CCGGAAGCGCCCGCCGGCGGGACCGAGGAACGGCCGCTTGCCCCTGAGGCCGCCCGGCCTCCCCGGGG
GAGTTCGACTGGTGGGGACTGGGTTCAAGCCCGTGCCCGCCGACCGCTCCTCCGGACGGAAATTCCGG
GCCCGGCCGGGGCTCGCTCGGGGTCGGTGGCGAATCTGTCGGCATTCCACCCGACCCGTCTTGAAACA
CGGACCAAGGAGTCTAACATGCGCGCGAGTCATTGGGCTGTACGAAACCCAAAGGCGCAGTGAAAGCG
AGGGCCGTCTCGGACTGGCCCAGGTGGGATCCCTCT---
CCCCCTCGGGGGTGGGGGCGCACCACCGGCCCGTCCCGTCTGCGTCGTCAGTGGGGCGGAGCAGGAGC
GTGCACGCTGGGACCCGAAAGATGGTGAACTATGCCTGAGTAGAACGAAGCCAGAGGAAACTCTGGTG
GAAGTCCGTAGCGATTCTGAC
>S_tecta
TTAAGCATATAACTAAGCGGAGGAAAAGAAACTAACAAGGATCCCCCCAGTAACGGCGAGTGAAGCGG
GAAGAGCCCAGCACCGAATCCCCCGGCGTCGCGTCGGCGGGAACTGTGGTGTGTGGGACGCCACCAGT
CGCGCATGCCGATGCCCAAGTCCTCCTGATCGAGGCTTCACCCAGAGCGGGTGTAAGGCCTTTAGCAG
GTACTCGGCTGCGCGGCCGCGAGCGTCTCAGGAGTCGGGTTGTTTGGGAATGCAGCCCAAAGCGGGTG
GTAAACTCCATCTAAGGCTAAATACCGGCACGAGTCCGATAGCGGACAAGTACCGTGAGGGAAAGTTG
AAAAGAACTTTGAAGAGAGAGTTCAAGAGTACGTGAAACCGCTCAGAGGTAAACGGGTGGATCCGCAA
AGTCGGCCCGCGAGATTCAGCGCGGCGGCCGGGCGCGCGCGCCGGGATGAGAGGACTCTCTGAGGACC
CTTCCCGGCGCGGTCGAGCGTCGCGTCGCCGCGTGCACTTTTCGCGGGCAGAGCGCCACGACCGGTTC
CTCGGCGGCCACACACCGGTGGCGGGAAGGTAGGTGCGTCGCTCGTCGATGCACTGTTACAGCCCGTC
CCGGAAGCGCCCGCCGACGGGACCGAGGAACGGCCGCTTGCCCTCGAGGCCGCCCGGCCTCCCCGGGG
GAGTTCGACTGGCGGGGACTGGGTTCAAGCCCGTGCCCGCCGACCGCTCCTCCGGACGGAAATTCCGG
GCCCGGCCGGGGCTCGCTCGGGGTCGGTGGCGAATCTGTCGGCATTCCACCCGACCCGTCTTGAAACA
CGGACCAAGGAGTCTAACATGCGCGCGAGTCATTGGGCTGTACGAAACCCAAAGGCGCAGTGAAAGCG
AGGGCCGTCTCGGACTGGCCCAGGTGGGATCCCCTCCGTCCGCCTCGCGGGGGGGGGCGCACCACCGG
CCCGTCCCGTCTGCGCCGTCAGTGGGGCGGAGCAGGAGCGTGCACGCTGGGACCCGAAAGATGGTGAA
CTATGCCTGAGTAGAACGAAGCCA-------------------------------------
>N_swatowensis_1
TTAAGCATATAACTAAGCGGAGGAAAAGAAACTAACAAGGATCCCCCCAGTAACGGCGAGTGAAGCGG
GAAGAGCCCAGCACCGAATCCCCCGGCGCGCCGCCGGCGGGAACTGTGGTGTGCGGGACGCCACCAGT
CGCGCGTGCCGGCGCCCAAGTCCTCCTGATCGAGGCTTCACCCGGAGCGGGTGTAAGGCCTTTGGCAG
GTGCCCGGCTGCGCGGCCGCGAGCGTCCCAGGAGTCGGGTTGTTTGGGAATGCAGCCCAAAGCGGGTG
GTAAACTCCATCTAAGGCTAAATACCGGCACGAGTCCGATAGCGGACAAGTACCGTGAGGGAAAGTTG
AAAAGAACTTTGAAGAGAGAGTTCAAGAGTACGTGAAACCGCCCAGAGGTAAACGGGTGGATCCGCGA
AGTCGGCCCGCGAAATTCAGCGCGGCGGTCGGGCGCGCGCGCCGGGAGTGGGGGATCCTTCGAGGACC
CCGCCCGGCGCGGCTCGGCGTCGCACCGCCGCGTGCACTTTTCGCGGGCGGAGCGCCACGACCGGTTC
CTCGGCGGACACACGCCGGCGGCGGGAAGGTAGGTGCGCCGCCCGTCGGCGCACTGTTACAGCCCGCC
CCGGAGGCGCCCGCCGAAGGGACCGAGGAACGGCCGCCAGCCCTCGAGGCCGCCCGGCCTCCCCGGGG
GAGTCCGACCGGCGGGGACTGGGTTCAAGCCCGTGCCCGCCGACCGCTCCCCCGCGCAG----

CCGGGCGTCGGCCGGGGCCGGCTCGGGGTCGGTGGCGAATCTGTCGGCATTCCACCCGACCCGTCTTG
AAACACGGACCAAGGAGTCTAACATGCGCGCGAGTCATTGGGCCGTACGAAACCCAAAGGCGCAGTGA
AAGCGAGGGCCGTCTCGGACCGGCCCAGGTGGGATCCCCTCGACCTCCTCGGAGGAGGGGGGCGCACC
ACCGGCCCGTCCCGTCCGCGCCGTCGGTGGGGCGGAGCAGGAGCGTGCACGCTGGGACCCGAAAGATG
GTGAACTATGCCTGAGTAGGACGAAGCCAGAGGAAACTCTGGTGGAAGTCCGTAGCGATTCTGAC

Supplementary Infromation 4.

Partition subset	Models for MrBayes	Models for IQ-TREE
CO1 Codon Position 1	GTR+I+G	GTR+I+G
CO1 Codon Position 2	HKY+I	HKY+I
CO1 Codon Position 3	HKY+G	TIM+G
16S	GTR+I+G	TRN+I+G
28S	GTR+I+G	GTR+I+G

Supplementary Information 5

The Bayesian phylogenetic tree inferred from a combined dataset of two mtDNA sequences (CO1 and 16S; 1129 bp). Numbers at the branch nodes represent Bayesian posterior probability and Ultrafast bootstrap value in IQtree. Red colored OTUs indicates Vietnamese amphibolids. Detailed settings of analyses are below.

Bayesian phylogeny

Applied evolutionary model

Partition	Model
16S	GTR+G
CO1 Codon Position 1	GTR+I+G
CO1 Codon Position 2	HKY+I
CO1 Codon Position 3	HKY+I+G

Analysis setting

Generations 1,000,000
Sample freq. 100
Burn-in 10%
All ESS is over 200.

ML phylogeny

Applied evolutionary model

Partition	Model
16S	GTR+G
CO1 Codon Position 1	GTR+I+G
CO1 Codon Position 2	HKY+I
CO1 Codon Position 3	HKY+I+G

Analysis setting

Partition model: edge-proportional -spp
Assessing branch supports: UFBoot -bb
Replicates: 5,000

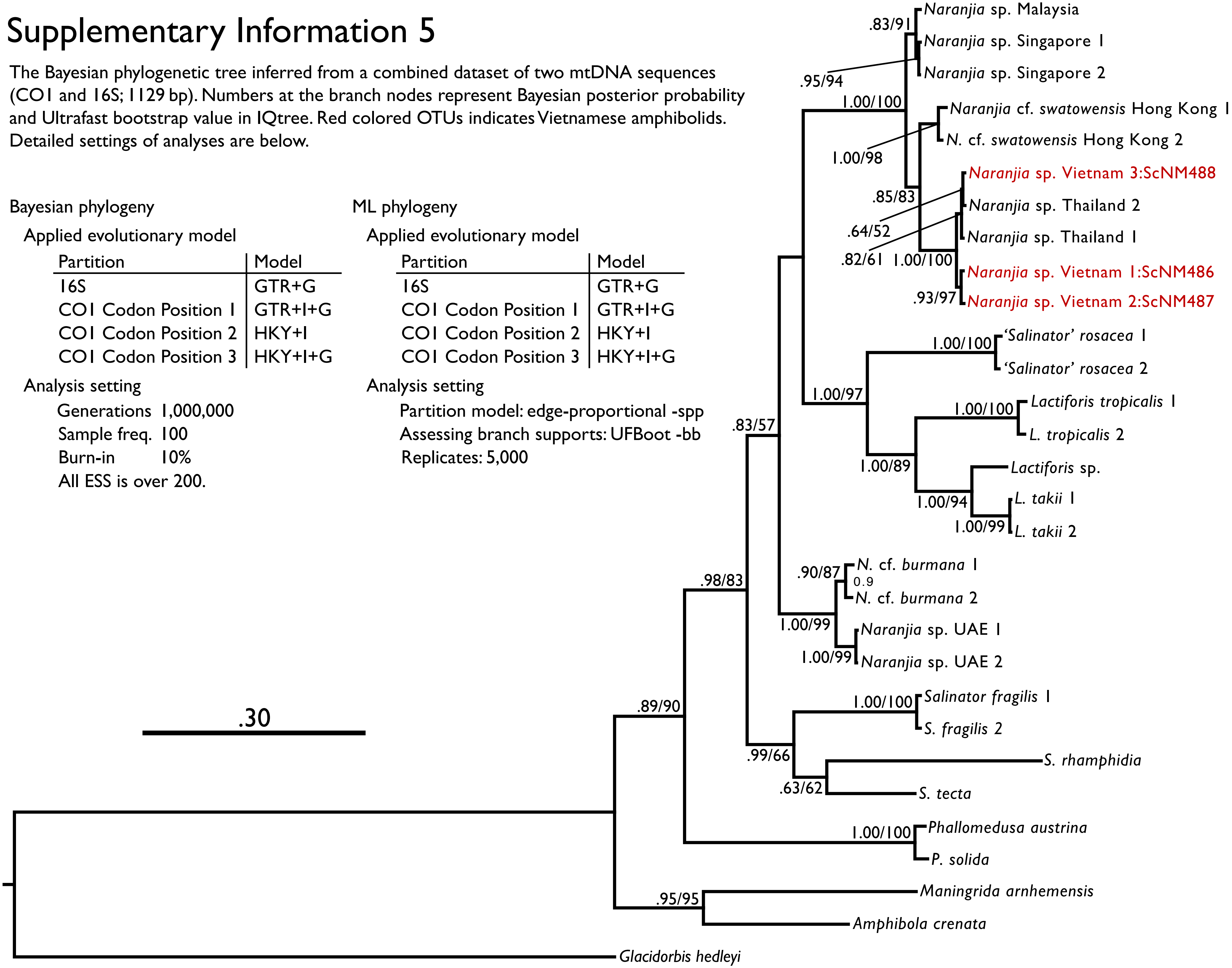

Supplementary Information 6

The Bayesian phylogenetic tree inferred from 28S sequences (1080 bp).
Numbers at the branch nodes represent Bayesian posterior probability and Ultrafast bootstrap value in IQtree.
Red colored OTUs indicates Vietnamese amphibolids.
Detailed settings of analyses are below.

Bayesian phylogeny

Applied evolutionary model

Partition	Model
28S	GTR+G

Analysis setting

Generations 1,000,000
Sample freq. 100
Burn-in 10%
All ESS is over 200.

ML phylogeny

Applied evolutionary model

Partition	Model
28S	GTR+G

Analysis setting

Partition model: edge-proportional -spp
Assessing branch supports: UFBoot -bb
Replicates: 5,000

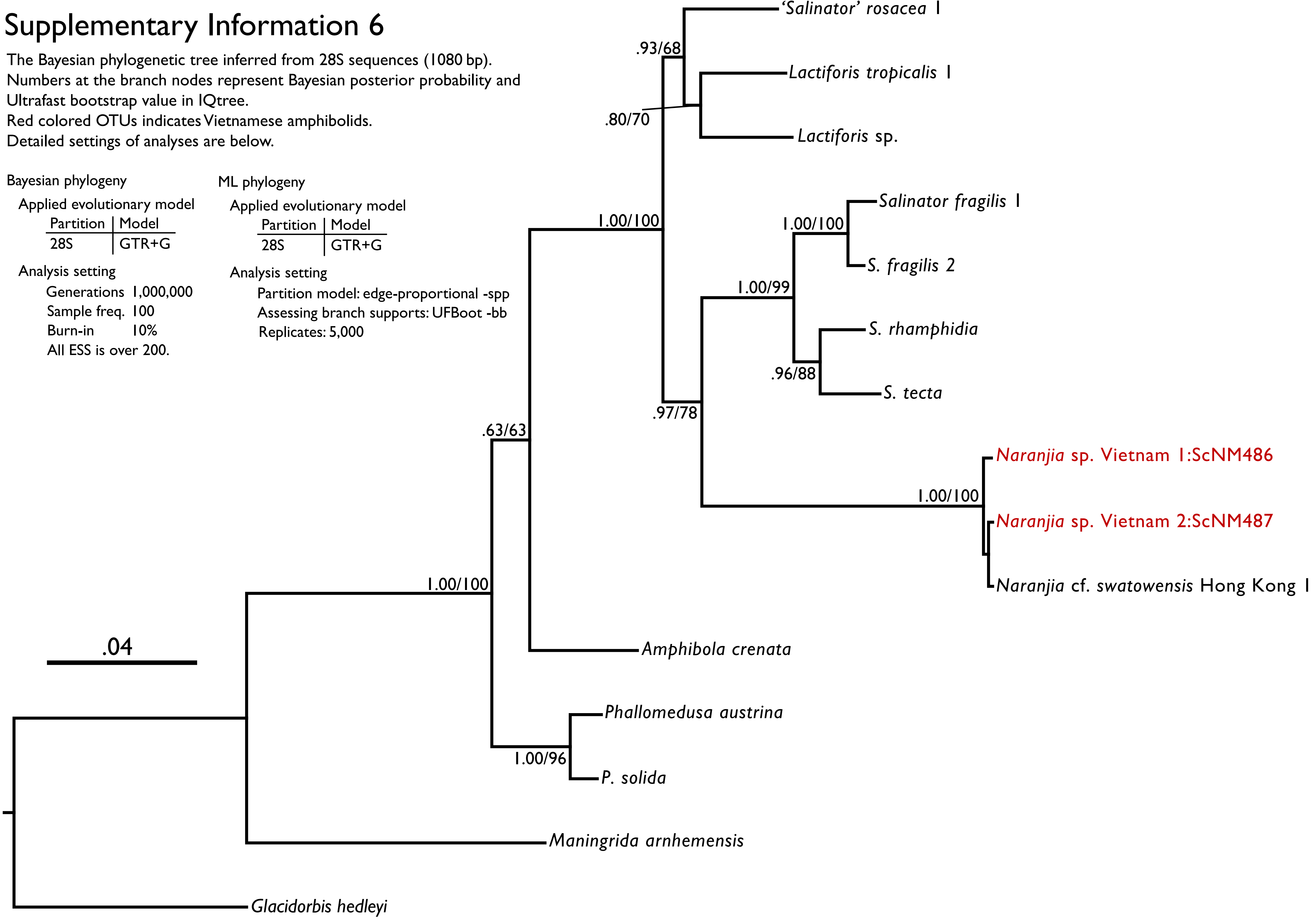

Supplementary Information 7

The shells of Amphibolidae in the museums.
a. *Amphibola quadrasi* of malacology collection in the Academy of Natural Science, Philadelphia (ANSP) (Cat. No. A3498). SL = 7.4 mm. b. "*Amphibola*" *burmana* of collection in ANSP (Cat. No. A3504). SL = 7.8 mm. c–e. The shells of "*Salinator*" *quadrasi* in University Museum Fujukan, University of the Ryukyus (Cat. No. RUMF-ZM-08183). SL = c: 6.75 mm, d: 8.7 mm, e: 10.02 mm.
The museum material consisted of several specimens. A selection of representative specimens is shown here. Materials a-b were photographed by K. Seizova, ANSP and edited by T. Saito. Materials c-e were photographed and edited by T. Saito.
Scale bar = 1 mm.

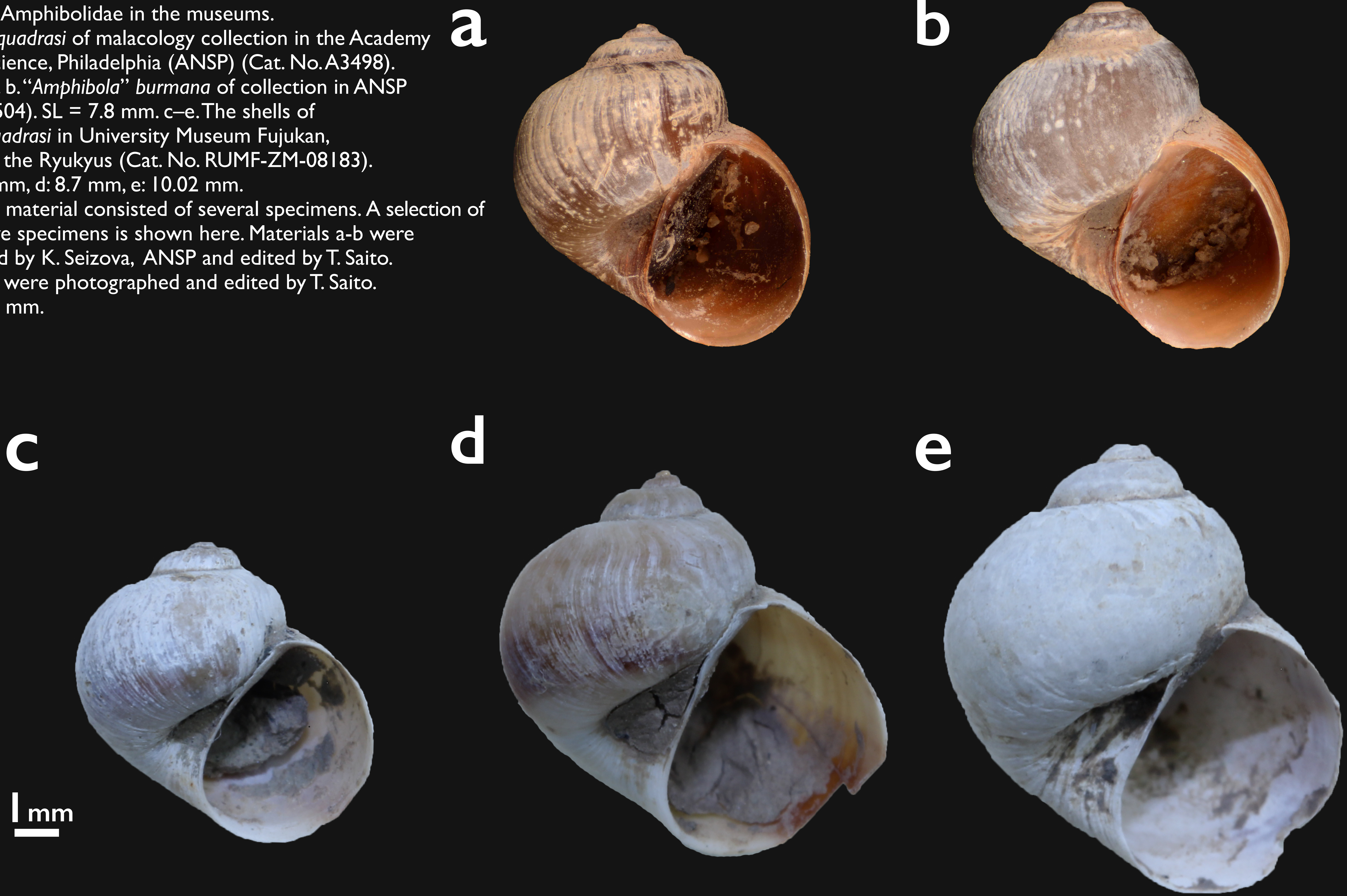

Supplement: Supplementary file 2 — Supplementary Information 2–7. [file 41598_2020_75200_MOESM2_ESM.pdf]
